# An Updated Checklist of the Sicilian Native Edible Plants: Preserving the Traditional Ecological Knowledge of Century-Old Agro-Pastoral Landscapes

**DOI:** 10.3389/fpls.2020.00388

**Published:** 2020-04-29

**Authors:** Salvatore Pasta, Alfonso La Rosa, Giuseppe Garfì, Corrado Marcenò, Alessandro Silvestre Gristina, Francesco Carimi, Riccardo Guarino

**Affiliations:** ^1^Institute of Biosciences and Bioresources (IBBR), National Research Council of Italy (CNR), Palermo, Italy; ^2^Cooperativa Silene, Palermo, Italy; ^3^Department of Botany and Zoology, Faculty of Science, Masaryk University, Brno, Czechia; ^4^Dipartimento STeBiCeF, Sezione Botanica, University of Palermo, Palermo, Italy

**Keywords:** ethnobotany, agro-pastoral landscapes, sustainable agriculture, TEK (traditional environmental knowledge), Ellenberg Indicator Values (EIV)

## Abstract

The traditional use of native wild food plants (NWFP) may represent a valuable supplementary food source for the present and future generations. In Sicily, the use of wild plants in the human diet dates back to very ancient times and still plays an important role in some rural communities. Moreover, in this regard, the natural and cultural inheritance of this island is wealthy and diversified for several reasons. First, Sicily hosts a rich vascular flora, with 3,000 native and 350 endemic plants. Second, due to its central position in the Mediterranean, the island has acted as a veritable melting pot for the ethnobotanical knowledge of the rural communities of the entire basin. We reviewed all the available literature and, starting from such omnicomprehensive checklist, partially improved thanks to the data issuing from recent field investigations, we critically revised the whole species list, basing our review on field data issuing from interviews and on our expert knowledge. As a result, we provide a substantially updated list of 292 NWFP growing on the island. Further 34 species, reported as NWFP on previous papers were discarded because they are not native to Sicily, while 45 species were listed separately because their identity, occurrence and local use as food is doubtful and needs to be further investigated. Moreover, we tried to shed light on the ecology (growth form and preferential habitat) of the Sicilian NWFP, with special focus on crop wild relatives (CWR). Our preliminary ecological analyses point out that a high percentage of these plants are linked with the so-called ‘cultural’ landscapes, patchy semi-natural environments rich in ecotones, leading to the conclusion that the maintenance of century-old agro-pastoral practices may represent an effective way to preserve the local heritage of edible plants. Our study allowed to identify as much as 102 taxa of agronomic interest which could be tested as novel crops in order to face ongoing global changes and to comply with sustainable agriculture policies. Among them, 39 taxa show promising traits in terms of tolerance to one or more environmental stress factors, while 55 more are considered CWR and/or can be easily cultivated and/or show high productivity/yield potential.

## Introduction

**FIGURE 1:**
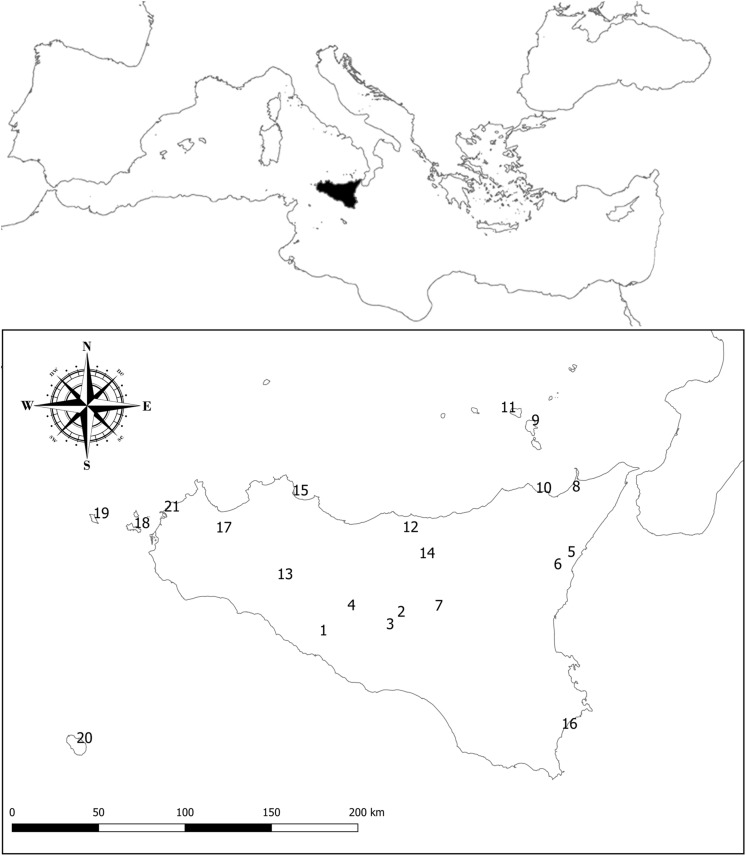
Sicily (black) and its position in the Mediterranean Basin. The numbers refer to the localities where interviews were carried out in order to perform an additional assessment of the harvest frequency of Sicilian NWFP and to update the available knowledge on their use (see Supplementary Table S1), i.e., Joppolo Giancaxio (1), Caltanissetta (2), San Cataldo (3), Sutera (4), Piedimonte Etneo (5), Sant’Alfio (6), Pergusa (7), Barcellona Pozzo di Gotto (8), Lipari Island (9), Patti (10), Salina Island (11), Castelbuono (12), Chiusa Sclafani (13), Gangi (14), Palermo (15), Avola (16), Calatafimi (17), Favignana Island (18), Marettimo Island (19), Pantelleria Island (20), and Trapani (21).

Sicily is the largest Mediterranean island (Figure 1), with an extension of c. 25,400 km^2^. Its territory is predominantly hilly or mountainous, with less than 20% of its surface below 300 m a.s.l. The geographical position of Sicily, its complex geological history (Carbone et al., 2018) and the sharp topographic, edaphic and climatic contrasts make this island one of the most heterogeneous Mediterranean territories. Moreover, Sicily and its satellite islands belong to the Tyrrhenian area, one of the main hot-spots of plant diversity in the whole Mediterranean basin (Médail and Quézel, 1999). Its rich flora counts about 3,000 native plant taxa, around 12% of which are endemics (Guarino and Pasta, 2018).

Thanks to its location, Sicily has also been a major pathway of human migration, acting as a cultural, economic and ethnic crossing point and melting pot. Human presence in Sicily knows no pauses since 14–13 thousand years ago (Mannino et al., 2012), and with no doubt during this long time-lapse entire plant assemblages have been profoundly shaped by hunters-gatherers, and subsequently even wiped out by the early onset of agro-silvopastoral practices (Leighton, 1999; Tinner et al., 2016).

The vegetation of the island shows almost everywhere the traces of long-lasting land exploitation; not surprisingly, anthropogenic plant communities (*Artemisietea vulgaris*, *Chenopodietea*, *Papaveretea rhoeadis*, *Parietarietea judaicae*, *Poetea bulbosae*, and *Polygono-Poetea*, etc.) characterize almost 50% of the island’s vegetation (Guarino and Pasta, 2017). Furthermore, hard-wheat crop fields currently occupy a large portion of the island’s territory, and other traditional forms of dry-land farming (olive, almond, carob tree, pistachio, ash-tree, hazelnut, and chestnut groves) still characterize part of the Sicilian rural landscape. Along with the disappearance of traditional practices (e.g., seasonal transhumance) and crafts (e.g., charcoal burners, pipe manufacturers, cork and sumac gatherers, miners, millers, etc.), also many man-made landscapes and habitats, such as dry-stone terraces, fruit orchards, dry-land groves and fallows are fading or have already disappeared. Abandonment and land-use changes are responsible for the fast collapse of local natural and cultivated plant richness (La Mantia et al., 2011; Albrecht et al., 2016). Currently, those traditional landscapes and many others suffer from abandonment or, worse, undergo deep transformations due to agricultural intensification and urban sprawl. Intensive cultivations already cover around 25% of the island’s surface, and they are still expanding. Two blatant examples are the ongoing replacement of *Citrus* orchards with greenhouses, while intensive vineyards are gradually substituting dry groves. Mechanized agricultural practices and the massive input of chemical fertilizers and pesticides select the weeds, to the detriment of Mediterranean plants and archaeophytes and favoring plenty of non-native tropical and subtropical species, which also take advantage of high nutrient- and water input (Guarino et al., 2015).

In respect to this scenario and considering the challenges related to future global climate changes, Sicilian native wild food plants (from now on NWFP) could play a significant role with respect to the fulfillment of both sustainable agriculture and dietary supplement. In addition to raising awareness on the importance of Mediterranean diet (Willett, 2006; Gamboni et al., 2012), there are many other good reasons to go on with the investigation and valuation of the ethnobotanical knowledge on NWFP throughout the Mediterranean area (Guijt, 1998). In fact, during the last 20 years, an increasing number of scientific papers (e.g., Grivetti and Ogle, 2000; Trichopoulou et al., 2000; Simopoulos, 2004; Pieroni and Price, 2005; Schaffer et al., 2005; Bharucha and Pretty, 2010; Ranfa et al., 2014; Disciglio et al., 2017; Renna, 2017) highlighted the strong correlation between wild food consumption and health.

An increasing amount of field data highlights the fact that the Mediterranean countries were not only important as a target of the spread of the so-called ‘Neolithic revolution’ but played an active role in the early cultivation of the wild ecotypes of several woody species, such as *Olea europaea* (Diez et al., 2015), *Ceratonia siliqua* (Viruel et al., 2019), and *Vitis vinifera* (De Michele et al., 2019), as well as of many vegetables (e.g., *Allium* spp., *Brassica* spp., *Cynara* spp., etc.) and cereal crops (e.g., *Avena* spp., *Hordeum* spp., and *Triticum* spp.), even far before than expected (Snir et al., 2015). The current change of paradigm about the history and geography of early agriculture underlines the importance of studying, conserving and effectively exploiting the germplasm of Mediterranean CWR.

In this paper, we aimed to critically review the so far available lists of traditional Sicilian NWFP, and to provide a first evaluation of the ecological requirements of these plants, paying special attention to the natural and semi-natural ecosystems where they grow and stressing the importance of managing and maintaining them in view of the ongoing global changes. These ecosystems represent important potential reservoirs of food because they host most of the NWFP and many CWR. Additionally, we evaluated some ecological features of NWFP that could be retained as traits of interest for the detection of novel crops and/or the improvement of the existing ones. These plants may help to face the future challenges of Mediterranean agriculture due to global change, such as extreme heat events, water shortage, salinization and adaptation to nutrient-poor soils.

## Materials and Methods

### Data Set

The list of the Sicilian NWFP provided by Pasta et al. (2011), based on a critical review of the whole regional ethnobotanical literature concerning edible plants up to 2010, was improved and updated by consulting all the most recent and authoritative works on this topic, i.e., Arcidiacono (2016), Schicchi and Geraci (2016), Morreale (2018), and Geraci et al. (2018). Coria (1989), Lentini and Mazzola (1998), Arcidiacono et al. (2007, 2010), as well as all the Sicilian papers published after 2010, i.e., Aleo et al. (2013), Tuttolomondo et al. (2014a, b), Mazzola et al. (2015), Signorello (2015), Licata et al. (2016), Quave and Saitta (2016), Schicchi and Geraci (2016), Cucinotta and Pieroni (2018), Gargano et al. (2018), and Monello (2018) were consulted, too.

The works of Ghirardini et al. (2007) and Guarrera and Savo (2016) were used as basic reference for the whole Italian territory. The assessment of the native status of the Sicilian edible plants was mostly based on Galasso et al. (2018), and non-native food plants were not taken into account for further elaborations. For each considered taxon, we reported the scientific name according to the second edition of the Flora of Italy (Pignatti et al., 2017–2019), the synonym(s) encountered in the consulted literature on NWFP, and the plant family according to Chase et al. (2016), while their growth form was assessed according to Pignatti et al. (2017–2019). We decided to skip the analysis of the life forms and chorotypes of the Sicilian NWFP and we provided just basic information concerning other interesting ethnobotanical issues, such as edible part(s) and traditional use(s) as food, because all these topics have been already treated in the most recent and comprehensive papers concerning Sicilian edible plants (Lentini and Venza, 2007; Pasta et al., 2011; Geraci et al., 2018). Basic information on the potential risks induced by toxic or poisonous compounds contained in the Sicilian NWFP is provided, too. Additionally, a preliminary semi-quantitative evaluation of the importance of Sicilian NWFP to local harvesters has been performed by comparing the data reported by Geraci et al. (2018) with those issuing from the interviews carried out across the whole Sicilian territory between 2016 and 2019 (Figure 1).

Finally, we annotated whether the listed NWFP were considered CWR by Heywood and Zohary (1995) or maybe deemed CWR following the definition: ‘A crop wild relative is a wild plant taxon that has an indirect use derived from its relatively close genetic relationship to a crop’ (Maxted et al., 2006).

### Ecological Assessment

In order to outline the ecological preferences of the Sicilian NWFP, the altitudinal range and the Ellenberg Indicator Values (EIVs) concerning each species, extracted from the database of Pignatti et al. (2017–2019), were adopted.

The preferential habitat types of the Sicilian NWFP were assessed following the list of diagnostic species of phytosociological classes of the European vascular plant communities (Mucina et al., 2016, Appendix S6). The phytosociological classes were associated with the EUNIS habitats recorded from Sicily in Carta della Natura (ISPRA, 2012) according to the crosswalk proposed by Angelini et al. (2009). In order to obtain more clues about the correlation between NWFP and different habitat types, we also used the habitats assigned in the database of Pignatti et al. (2017–2019), which recognizes only 24 habitat types belonging to three main ecological groups, i.e., ‘terrestrial,’ ‘aquatic,’ and ‘anthropogenic.’ In both cases, all the habitats where a given species may occur were assigned according to the fuzzy logic, with a numerical score ranging from 0 to 1. In this way, the fuzzy value was equal to 1 for the stenoecious species, whereas for the dioecious species the fuzzy values resulted to be >0 and <1 depending on the number of habitats assigned to a species.

We used the non-metric multidimensional scaling (NMDS) analysis to summarize the similarity in habitat composition among NWFP, using Bray–Curtis distance measure to calculate the NWFP distance. This analysis was performed by CANOCO 5 (ter Braak and Smilauer, 2012).

We avoided to correlate the values concerning the relative abundance of edible plants basing on the habitat coverage reported in the already mentioned Sicilian map of EUNIS habitats because we considered that any attempt to do this without a statistically significant amount of plot data may lead to unreliable and even misleading results. In fact, a plant may be very rare or very frequent in a given habitat regardless to the area of occupancy of the habitat itself in the island.

## Results

### Inventory of Sicilian NWFP

A critical and updated inventory is provided and counts a total of 292 Sicilian NWFP (Supplementary Table S1). The taxa are listed in alphabetical order according to their scientific name. Synonyms, plant family, growth form, EIVs, altitudinal range, preferential habitat types, and CWR status are reported. Basic information on edible part(s), raw vs. cooked consumption, potential risks due to toxic or poisonous compounds, importance to local harvesters are provided, too.

According to the data issuing from our field investigations and those published by Geraci et al. (2018), the most commonly harvested Sicilian NWFP are *Asparagus acutifolius*, *Beta vulgaris* subsp. *maritima*, *Borago officinalis*, *Brassica rapa* subsp. *campestris*, *Cichorium intybus*, *Foeniculum vulgare s.l*., *Sonchus oleraceus* and *Sonchus tenerrimus*, while the less commonly harvested are *Centranthus ruber*, *Narcissus tazetta s.l*., *Papaver setigerum*, *Rorippa sylvestris*, *Rumex crispus*, and *Tordylium apulum*.

Our inventory includes 28 species which should probably be considered as archaeophytes in Sicily, but they are not mentioned in the checklist of the vascular alien flora of Italy (Galasso et al., 2018), and their regional status remains uncertain. The scientific name of these plant taxa is followed by an asterisk in the Supplementary Table S1. Moreover, many species commonly cultivated in Sicily and sometimes spreading from cultivations into semi-natural habitats were also taken into account, because palaeobotanical records testify that they occurred in Sicily throughout the Holocene. Hence, their native status cannot be discarded, even if their current distribution does not mirror the distribution range of their original wild populations. This is the case of *Castanea sativa*, *Celtis australis*, *Ceratonia siliqua*, *Corylus avellana*, *Ficus carica*, *Laurus nobilis*, *Mespilus germanica*, *Olea europaea*, *Pinus pinea*, *Sorbus domestica*, and *Vitis vinifera*.

On the contrary, a set of 34 plant species, despite being mentioned in the Sicilian ethnobotanical literature, were not considered in our analyses because they are not native (23 archaeophytes and 11 neophytes, see Supplementary Table S2). We also treated as archaeophytes several species which are native to other Italian regions but have certainly been introduced by man in Sicily. This is the case of *Asparagus officinalis*, *Rhus coriaria*, *Ruscus hypophyllum*, and *Salvia officinalis*, never spreading far from rural and disturbed areas, *Origanum onites*, whose Sicilian distribution range is restricted to the ruins of the ancient Syracuse, and *Cercis siliquastrum*, recently experiencing some successful colonization even in semi-natural habitats, but always next to the urban areas where it has been introduced.

The list of Sicilian NWFP was revised taking into account also the misleading information contained in some previous studies. In fact, due to identification errors, to the misinterpretation of Sicilian vernacular plant names and/or to the inappropriate inclusion of congeneric species, some authors reported plants which, either: (1) do not occur in Sicily or at least not in the study areas the papers were focused on; (2) have an extremely narrow niche and distribution range, so they could not be considered of any interest for local rural communities; (3) have been assumed to be food plants because they belong to the same genus of some NWFP, but their local exploitation needs to be better documented; (4) sometimes are used in Sicily as healing plants but cannot be used raw as food because they contain stinky, disgusting, toxic or even lethal compounds.

As a result of all the considerations above, 45 wrong, questionable and doubtful records were excluded from our NWFP inventory. These are listed in Supplementary Table S3, along with the reasons for the rejection and the source(s) of the wrong/doubtful records.

Indeed, numerous reliable sources attest that Sicilian people harvest and eat at least 30 potentially toxic and 11 poisonous plant species. The Apiaceae (11 taxa) and Polygonaceae (6) account for more than 40% of them. Other families rich in dangerous food plants are Amaryllidaceae, Asteraceae, Fabaceae, and Ranunculaceae (3 taxa each), and Asphodelaceae, Iridaceae, and Solanaceae (2).

### Diversity and Ecology of Sicilian NWFP

**FIGURE 2:**
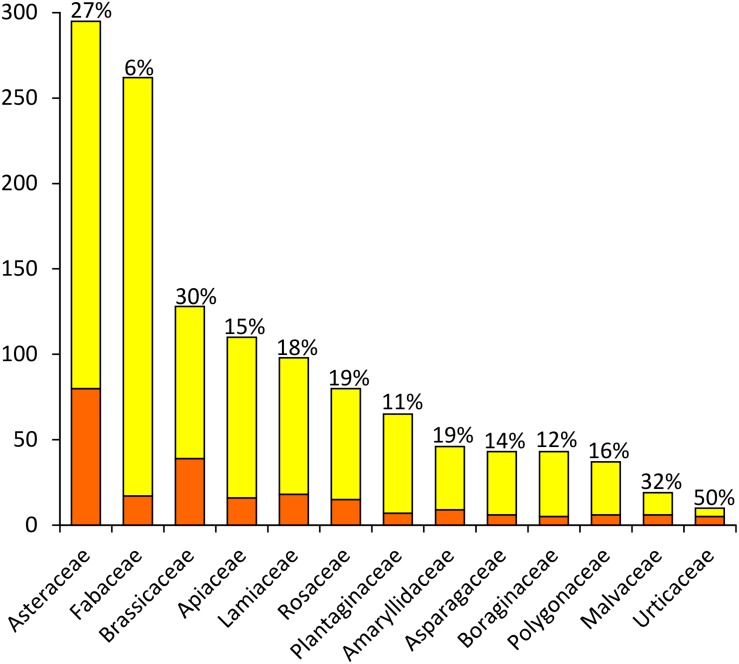
Plant families including more than 5 NWFP in Sicily. Orange: number of NWFP; Yellow: number of non-edible plants. The percentage of NWFP per family is reported on the top of each column.

The 292 taxa listed in Supplementary Table S1 belong to 51 plant families; Figure 2 shows the families including more than 5 NWFP. Around 2/3 of the Sicilian NWFP belong to six families only, i.e., Asteraceae (80, i.e., 27.4% of the whole list), Brassicaceae (39, 13.4%), Lamiaceae (18, 6.2%), Fabaceae (18, 6.1%), Apiaceae (16, 5.5%), and Rosaceae (16, 5.5%). The number of species and genera mirrors the overall taxonomic richness of the main NWFP families, except for Fabaceae, which showed rather a low percentage of edible species despite being one of the richest families of the Sicilian vascular flora. None of the Sicilian NWFP belongs to Poaceae, even if this family is the second richest of the Sicilian vascular flora, counting 293 different species on the Island.

**FIGURE 3:**
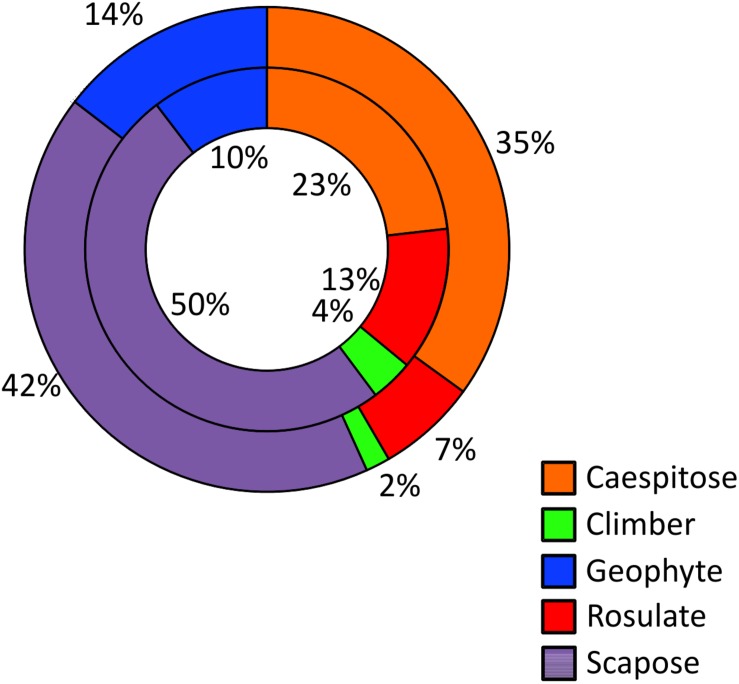
Frequency of the five commonest growth forms among NWFP (inner circle) and in the entire Sicilian vascular flora (outer circle).

If we focus on the five growth forms which count more than 10 Sicilian NWFP, the rosulate edible herbs result proportionally more represented than in the whole regional flora and the same pattern has been observed for climbing herbs and lianas (Figure 3). The percentage of scapose species among wild food plants is slightly higher, whereas that of caespitose and geophytes slightly lower than that of the whole Sicilian flora.

**FIGURE 4:**
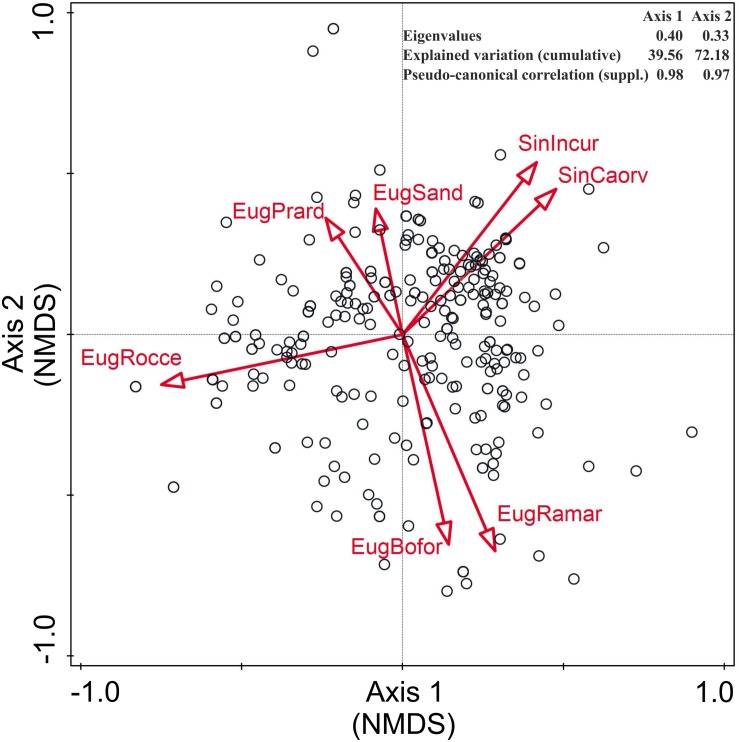
Non-metric multidimensional scaling (NMDS) ordination plot of the NWFP of Sicily with respect to the habitats reported in the Flora of Italy (Pignatti et al., 2017–2019). Only the habitats explaining >3% of the total variance are displayed. EugBofor, woodlands and forests; EugPrard, dry grasslands; EugRamar, edges, clearings, deciduous shrubberies; EugRocce, rocks, barks, small outcrops; EugSand, sands; SinCaorv, crops, vegetable gardens, orchards, vineyards, olive groves; and SinIncur, fallows, ruderal and urban habitats.

The relationship between the NWFP and habitat types is shown in the NMDS ordination plots (Figure 4 and Supplementary Figure S1). In both of the considered habitat classifications, most species are concentrated near the center of the graphics, proving to be rather dioecious, with a clear preference for anthropogenic habitats. The first two axes of the NMDS explain 80.7% (EUNIS) and 72.18% (Flora of Italy) of the total variance, and in both cases, they appear to be related to a gradient of anthropogenic disturbance and a gradient of edaphic humidity, respectively.

Non-metric multidimensional scaling ordination plots show the prevalence of ruderal-nitrophilous species linked to anthropogenic vegetation and man-made habitats and ecotones, which represent the core of local edible plants. Yet, three further species groups cluster rather clearly: (1) the fruit-bearing woody species linked to forest and shrubland communities, (2) a group rich in chamaephytes and hemicryptophytes adapted to the harsh conditions of bare and nutrient-poor soils mostly occurring in rocky habitats, and (3) a group including many species adapted to sandy or salty soils.

### Good Performers and Stress-Tolerant Sicilian NWFP

As far as EIVs are concerned, some significant differences in the *U*-, *R*-, and *N*-values of the Sicilian NWFP with respect to the whole regional flora (data not shown) have been detected. In particular, even if the mean values are comparable, the EIVs assigned to the NWFP tend to be less dispersed than those of the regional flora. Based on the EIVs, the Sicilian NWFP displaying the maximum tolerance to heat, drought, soil salinity and adaptation to nutrient-poor soils are reported in Table 1. This list includes 39 plant species having good performances under harsh environmental conditions, being able to overcome at least one out of four severe stress factors, i.e., two edaphic (low soil nutrient content and high soil salinity) ad two climatic (lack of water and extreme thermic events). In particular, *Capparis* species can be considered well adapted to harsh climatic and edaphic conditions (Mercati et al., 2019), with *Capparis sicula* showing a high ecological plasticity, as it can survive both in poorly aerated, salty and clayey soils as well as in secondary habitats such as roadsides (Gristina et al., 2014), while *C. spinosa* mainly colonizes coastal rocky cliffs. Wild cabbages (*Brassica* spp.) can thrive on nutrient-poor soils and tolerate intense heat and water stress growing on base-rich cliffs (from the coastline up to 1,000–1,200 m a.s.l.), while *Diplotaxis crassifolia* and *Thymbra capitata* can stand the enduring summer drought stress typical to Mediterranean grasslands and shrublands. Many NWFP belonging to Asteraceae, Brassicaceae, Lamiaceae, Valerianaceae tolerate very low soil nutrient availability and water stress but cannot stand thermal stress. *Cakile maritima*, *Crithmum maritimum*, *Echinophora spinosa*, and *Juncus acutus*, all growing in coastal habitats, are well adapted to salt-rich soils, the last two being resistant to nutrient-poor soils and only the latter also tolerant to water stress.

**TABLE 1:** An overview on the Sicilian NWFP which show the best performance with respect to four major stress factors, as suggested by the numerical values of the selected EIVs, i.e., T (temperature, range 1–12), U (edaphic humidity, range 1–11), N (nutrients, range 1–9), and S (salinity, range 0–3).

**Scientific name according to Pignatti et al. (2017–2019)**	**Plant Family (Chase et al., 2016)**	***T***	***U***	***N***	***S***
Taxa which are well adapted to climatic and edaphic stress					
*Capparis sicula* Veill.	Capparaceae	10	2	**1**	**3**
*Capparis spinosa* L.	Capparaceae	10	2	**1**	2
*Lotus cytisoides* L.	Fabaceae	10	**1**	**1**	2
Taxa which are well adapted to nutrient-poor soils and tolerate thermic and water stress					
*Astragalus boeticus* L.	Fabaceae	11	**1**	**1**	0
*Biscutella maritima* Ten.	Brassicaceae	10	2	**1**	0
*Brassica incana* Ten.	Brassicaceae	10	2	**1**	0
*Brassica rupestris* Raf.	Brassicaceae	10	2	**1**	0
*Brassica tournefortii* Gouan	Brassicaceae	**12**	2	**1**	0
*Carlina gummifera* (L.) Less.	Asteraceae	11	**1**	**1**	0
*Carlina sicula* Ten.	Asteraceae	11	**1**	**1**	0
*Chamaerops humilis* L.	Arecaceae	10	**1**	**1**	0
*Diplotaxis crassifolia* (Raf.) DC.	Brassicaceae	11	2	**1**	0
*Plantago serraria* L.	Plantaginaceae	10	2	**1**	0
*Rumex bucephalophoru*s L.	Polygonaceae	**12**	2	**1**	0
*Thymbra capitata* (L.) Cav.	Lamiaceae	10	2	**1**	0
Taxa which are well adapted to nutrient-poor and salt-rich soils and tolerant to water stress					
*Crithmum maritimum* L.	Apiaceae	8	**1**	**1**	**3**
Taxa which are adapted to nutrient-poor and salt-rich soils					
*Echinophora spinosa* L.	Apiaceae	8	4	**1**	2
Taxa which are well adapted to nutrient-poor soils and tolerant to water stress					
*Centranthus ruber* (L.) DC.	Valerianaceae	8	2	**1**	0
*Fedia graciliflora* Fisch. & C.A. Mey.	Valerianaceae	9	2	**1**	0
*Hyoseris radiata* L.	Asteraceae	8	2	**1**	0
*Hyoseris scabra* L.	Asteraceae	9	**1**	**1**	0
*Hypochoeris cretensis* (L.) Chaub. & Bory	Asteraceae	8	2	**1**	0
*Hypochaeris glabra* L.	Asteraceae	8	2	**1**	0
*Hypochaeris laevigata* (L.) Ces., Pass. & Gibelli	Asteraceae	8	2	**1**	0
*Hypochaeris radicata* L.	Asteraceae	8	2	**1**	0
*Lavandula stoechas* L.	Lamiaceae	9	2	**1**	0
*Lobularia maritima* (L.) Desv.	Brassicaceae	9	2	**1**	0
*Micromeria juliana* (L.) Benth. ex Rchb.	Lamiaceae	8	2	**1**	0
*Papaver setigerum* DC.	Papaveraceae	8	2	**1**	0
*Phagnalon saxatile* (L.) Cass.	Asteraceae	9	2	**1**	0
*Rosmarinus officinalis* L.	Lamiaceae	8	2	**1**	0
*Thymus spinulosus* Ten.	Lamiaceae	8	2	**1**	0
*Tolpis umbellata* Bertol.	Asteraceae	9	2	**1**	0
*Tolpis virgata* (Desf.) Bertol.	Asteraceae	8	2	**1**	0
*Valerianella eriocarpa* Desv.	Valerianaceae	9	2	**1**	0
Taxa which are well adapted to nutrient-poor soils					
*Plantago lagopus* L.	Plantaginaceae	9	3	**1**	0
*Reseda alba* L. s.l.	Resedaceae	8	3	**1**	0
Taxa which are adapted to salt-rich soils					
*Cakile maritima* Scop.	Brassicaceae	8	6	8	**3**
*Juncus acutus* L.	Juncaceae	8	8	3	**3**
**Number of taxa showing the maximum value of tolerance to the selected stress factors**		**2**	**7**	**37**	**4**

*Best responses to one or more stress factors are in bold.*

### Sicilian NWFP and CWR

Based on literature reviews, we found that as much as 55 Sicilian NWFP (i.e., 18.8% of the total) are CWR (Table 2). Many of these plant taxa show high interest for their promising agronomic traits and/or for their high stress-tolerance. The richest families in CWR are Brassicaceae (9 taxa), Rosaceae (8), Asteraceae (7), Lamiaceae (6), Fabaceae (5), Apiaceae, and Asparagaceae (4), Amaryllidaceae and Capparaceae (2). With four taxa, the genera *Asparagus* and *Brassica* result to be the richest ones, followed by *Allium*, *Capparis*, *Cichorium*, *Mentha*, *Rubus*, *Salvia*, and *Sorbus* (2 taxa each).

**TABLE 2:** Families of Sicilian NWFP – including CWR and/or stress-tolerant taxa – to be tested in order to detect and develop new crops for their high potential as resources for agronomic, genetic, pharmaceutical, and nutritional purposes.

**Family of NWFP of agronomic interest**	**Number of sp./subsp.**	**Number of genera**	**Number of CWR**	**CWR**	**Other taxa to be tested for the development of new crops**	**Other taxa to be tested for the development of new stress-tolerant crops**
**Amaranthaceae**	2	2	1	*Beta vulgaris* L. subsp. *maritima*(L.) Arcang.	*Chenopodium album* L.	
**Amaryllidaceae**	4	1	2	*Allium ampeloprasum* L. *Allium commutatum* Guss.	*Allium ursinum* L. *Allium triquetrum*L.	
**Apiaceae**	6	6	4	*Apium graveolens* L. *Daucus carota* L. *s.l*. *Foeniculum vulgare* Mill. *s.l*. *Helosciadium nodiflorum* (L.) W.D.J. Koch subsp. *nodiflorum*		*Crithmum maritimum* L. *Echinophora spinosa* L.
**Asparagaceae**	6	3	4	*Asparagus acutifolius* L. *Asparagus albus* L. *Asparagus aphyllus* L. *Asparagus horridus* L.	*Muscari comosum* (L.) Mill. *Ruscus aculeatus* L.	
**Asteraceae**	29	19	7	*Cichorium intybus* L.*Cichorium pumilum*Jacq.*Cynara cardunculus* L. *s.l*.*Lactuca serriola* L.*Matricaria chamomilla* L.*Scorzonera undulata*Vahl subsp. *deliciosa* (DC.) Maire*Tragopogon porrifolius* L.	*Hedypnois cretica* (L.) Dum.Cours.*Hedypnois rhagadioloides* (L.) F.W.Schmidt*Helminthotheca echioides* (L.) J.Holub*Leontodon siculus* (Guss.) Nyman*Leontodon tuberosus* L.*Reichardia picroides* (L.) Roth*Rhagadiolus stellatus* (L.) Gaertn.*Scorzoneroides cichoracea* (Ten.) Greuter*Scorzoneroides muelleri* (Sch.Bip.) Greuter & Talavera subsp. *muelleriSonchus oleraceus* L.*Sonchus tenerrimus* L.*Urospermum picroides* (L.) F.W.Schmidt	*Carlina sicula* Ten.*Hyoseris radiata* L.*Hyoseris scabra* L.*Hypochaeris cretensis* (L.) Chaub. & Bory*Hypochaeris glabra* L.*Hypochaeris laevigata* (L.) Ces., Pass. & Gibelli*Hypochaeris radicata* L.*Phagnalon saxatile* (L.) Cass.*Tolpis umbellata* Bertol.*Tolpis virgata* (Desf.) Bertol. *s.l*.
**Betulaceae**	1	1	1	*Corylus avellana* L.		
**Brassicaceae**	17	11	9	*Barbarea bracteosa* Guss.*Brassica incana* Ten.*Brassica nigra* (L.) Koch*Brassica rapa* L. subsp. *campestris* (L.) Clapham*Brassica rupestris* Raf. *s.l*.*Eruca sativa* Mill.*Nasturtium officinale*R.Br.*Raphanus raphanistrum* L. *s.l*.*Sinapis alba* L. *s.l*.	*Erucastrum virgatum* C.Presl*Rapistrum rugosum* (L.) All.*Diplotaxis erucoides* (L.) DC.*Diplotaxis muralis* (L.) DC.*Diplotaxis tenuifolia* (L.) DC.	*Cakile maritima* Scop.*Lobularia maritima* (L.) Desv.*Diplotaxis crassifolia* (Raf.) DC.
**Capparaceae**	2	1	2	*Capparis sicula* Veill.*Capparis spinosa*L. subsp. *rupestris* (Sm.) Nyman		
**Ericaceae**	1	1	0		*Arbutus unedo* L.	
**Fabaceae**	4	4	4	*Ceratonia siliqua* L.*Glycyrrhiza glabra* L.*Lathyrus cicera* L.*Pisum sativum* L. subsp. *biflorum* (Raf.) Soldano*Vicia sativa* L.		
**Fagaceae**	1	1	1	*Castanea sativa* Mill.		
**Iridaceae**	1	1	1	*Crocus longiflorus* Raf.		
**Lamiaceae**	11	9	6	*Mentha spicata* L.*Mentha suaveolens* Ehrh.*Origanum vulgare* L. subsp. *viridulum* (Martrin-Donos) Nyman*Rosmarinus officinalis* L.*Salvia fruticosa* Mill. subsp. *thomasii*(Lacaita) Brullo, Guglielmo, Pavone, and Terrasi*Salvia sclarea* L.	*Clinopodium nepeta* (L.) Kuntze	*Lavandula stoechas* L.*Micromeria juliana* (L.) Benth. ex Rchb.*Thymbra capitata* (L.) Cav.*Thymus spinulosus* Ten.
**Lauraceae**	1	1	1	*Laurus nobilis* L.		
**Moraceae**	1	1	1	*Ficus carica* L. *s.l*.		
**Oleaceae**	1	1	1	*Olea europaea* L. var*. sylvestris* (Mill.) Lehr.		
**Rosaceae**	8	6	8	*Crataegus monogyna* Jacq.*Fragaria vesca* L.*Prunus spinosa* L.*Pyrus spinosa* Forssk.*Rubus idaeus* L.*Rubus ulmifolius* Schott*Sorbus aucuparia* L. subsp. *praemorsa* (Guss.) Nyman*Sorbus domestica* L.		
**Urticaceae**	4	1	0		*Urtica dioica* L.*Urtica membranacea* Poir.*Urtica pilulifera* L.*Urtica urens* L.	
**Valerianaceae**	1	1	1	*Valerianella locusta* (L.) Laterr.		
**Vitaceae**	1	1	1	*Vitis vinifera* L. subsp. *sylvestris* (C.C.Gmel.) Hegi		
**Total**	102	72	55			

To the family Asteraceae belong the wild progenitors of globe artichoke (*Cynara scolymus* L.), lettuce (*Lactuca serriola* L.) and chicories (*Cichorium intybus* and *Cichorium pumilum*), while the family Brassicaceae includes different species of wild cabbages (*Brassica incana*, *B. nigra*, *B. rupestris* and *B. tournefortii*) and wild rockets (*Eruca sativa*). Lamiaceae count many relatives of common spices such as mints (*Mentha spicata* and *Mentha suaveolens*), oregano (*Origanum vulgare* subsp. *viridulum*), rosemary (*Rosmarinus officinalis*), sages (*Salvia fruticosa* subsp. *thomasii* and *Salvia sclarea*). Other noteworthy CWR are the wild fennel (*Foeniculum vulgare*), wild strawberry (*Fragaria vesca*), wild garlic (*Allium ampeloprasum* and *Allium commutatum*) and wild capers (*Capparis sicula* and *C. spinosa*).

## Discussion

### A Matter of Natural and Land-Use History

The landscapes of Sicily are the result of anthropogenic disturbances occurring over several millennia. Through the centuries, rural communities have managed their environment and farmed the land in their natural way, creating a rich diversity of landscapes, choral representation of the historical identity of the territory and human cultural heritage (Guarino and Pasta, 2017, and references therein). One of the first effects of human land use was an increased fire frequency: wildfires were the easiest way to obtain grass-dominated terrains, which could be used as rangelands or, eventually, cultivated. The prevalence of NWFP in semi-natural and anthropogenic habitats could be seen as the result of a process of selection and adaptation lasting since 10,000 years, at least. The plants growing in habitats of this kind are providing most of the harvest, because it is easy to identify them and because they are usually found in dense and homogeneous populations. For instance, the relatively high percentage of rosulate plants among NWFP could mirror their ecological adaptation to grazing disturbance. This growth form enables to reduce the damages (namely defoliation) caused by the bites and by the trampling activity of domestic herbivores (Xu et al., 2013, and references therein). Most of the edible climbers, vines and lianas (e.g., *Clematis*, *Lathyrus*, *Rubia*, *Smilax*, *Tamus*, and *Vicia*) are instead common in ecotones, which have been created and shaped by humans.

As far as we know, no literature data is explaining why rosette-bearing and scapose (erect) taxa are more common than caespitose among wild food plants, whereas the relatively low percentage of edible geophytes is likely to depend on the high frequency of poisonous species within this group. As for EIVs, the values of ‘U’ (edaphic humidity) of the Sicilian edible plants appear to be relatively low. This pattern matches with the fact that basing on the bioclimatic classification of Sicily (Bazan et al., 2015), most of the Sicilian NWFP thrive under thermo-mediterranean bioclimatic conditions, characterized by long-lasting summer drought stress. The values of ‘R’ and ‘N’ indicate that many NWFP prefer base- and nutrient-rich soils, typical to the Sicilian hilly landscapes prone to human (namely agro-pastoral) practices. Interestingly, the high rate of ruderal-nitrophilous species among the edible plants appreciated for their fleshy stem or foliage was confirmed in the study carried out in northern Croatia by Vitasović Kosić et al. (2017), who also used EIVs to investigate the ecology of local wild food plants. Moreover, the altitudinal distribution pattern of the Sicilian NWFP (data not shown) suggest that they are slightly more concentrated in the lowlands and hilly areas of the island, where most of the permanent settlements and arable lands are concentrated since millennia.

Non-metric multidimensional scaling provides interesting clues on the spatial and temporal distribution of the food resources afforded by NWFP. For instance, most of the woody species are linked to forests, riverine plant communities and shrublands. They produce large amounts of fruits or berries that can be eaten raw, and represent an important food resource between the end of spring and the end of summer when all the annual and most of the perennial herbaceous NWFP are not available anymore.

Most of the edible Lamiaceae group together and are well adapted to nutrient-poor and rocky habitats (Figure 4 and Supplementary Figure S1), where they cope with water and nutrient shortage by producing many aromatic compounds that play the twofold action of helping them to save water and to inhibit predation by herbivores. Other stress-tolerant plants typical to sandy and/or salty soils form another cluster rich in species adapted to high salt input and long-lasting water shortage.

Under an ecological perspective, nutrient-poor habitats promote selection for traits allowing efficient resource conservation, while nutrient-rich environments select for species with acquisitive trait profiles (Reich, 2014). Domesticated plants, and in particular herbaceous crops, share few common traits and represent a small portion of the phenotypic spectrum displayed by their wild ancestors: the majority of those living on nutrient-rich soils bear soft, large and short-lived leaves, are fast-growing and proficient competitors, with high leaf nitrogen concentration and tall canopies (Freschet et al., 2015; Milla et al., 2018). As cultivation generally involved higher and more regular nutrient and water supply rates, humans probably focused their interest on NWFP bearing resource-acquisition trait profiles (Bogaard et al., 2013; Araus et al., 2014). Some of them, like the ancestors of several cereal crops, were chosen because they showed a rapid shift of their functional traits under domestication, being able to invest more energy on leaf biomass and height growth than other wild grasses that were used by hunter-gatherers, but were never domesticated (Cunniff et al., 2014). Many other cultivated plants descend from wild ancestors which were pre-adapted for cultivation thanks to several favoring traits (Triboullois et al., 2015; Martin-Robles et al., 2019). These considerations fit perfectly with the fact that most of the Sicilian NWFP, commonly used as vegetables and concentrated near the center of Figure 4 and Supplementary Figure S1, share many morphological and ecological traits: they colonize nutrient-rich soils and ecotones, they bear large and tender leaves, they grow very fast, they are good competitors.

The massive number of edible Asteraceae (subfam. Cichorioideae) is a common pattern in Mediterranean countries (Della et al., 2006; Leonti et al., 2006; Rivera et al., 2006; Hadjichambis et al., 2008). Interestingly, Cichorioideae appear to be strongly associated with pastoral activities since ancient times (Florenzano et al., 2015). Also, Brassicaceae are an essential component in wild harvesting in south Italian regions (Biscotti et al., 2018). These plants have probably been gathered since the very first stages of agriculture by Neolithic farmers, as they behaved as weeds.

All these ecological clues lead to the same conclusion: the majority of the Sicilian NWFP may be considered as ‘old companions’ of local human communities. Early Sicilians probably discovered very soon that many ‘proto-weeds’ colonizing the open spaces created after the disruption of pristine woodlands were useful and tasty, as it has been recently shown also for Israel (Snir et al., 2015). Also, the available palaeoecological studies (Sadori et al., 2008; Stika et al., 2008; Noti et al., 2009; Tinner et al., 2009) suggest that from the beginning of the Holocene (∼10 Ka) onward, human impact has been an important – if not the main – factor inducing the final opening of the Sicilian landscape. With their activities (burning, clearing, cutting, farming, plowing, etc.) local Neolithic communities not only fostered the success of many non-native pioneer light-demanding plants inadvertently introduced with crop species (the so-called ‘archaeophytes’) but also ended the shaping of the regional natural and semi-natural landscape, giving rise to a complex mosaic of prevalently open habitats with scattered nuclei of woodlands, shrublands and species-rich garrigues and grasslands (Guarino et al., 2005; Guarino, 2006; Brullo and Guarino, 2007).

### The Importance of a Multidisciplinary Approach

An unexpected result of our investigation concerned the detection of 46 wrong, questionable and doubtful plants that cannot be included among the Sicilian NWFP.

There are two distinct groups of experts coping with useful plants, plant uses and plant names: from one side the ‘practitioners,’ such as farmers, shepherds, artisans, cooks, forest workers, fishermen, hunters, etc., from the other side the ‘scholars,’ like ethnobotanists, ethno-anthropologists, philologists, historians, biochemists, veterinaries, etc. Until recent past, these two groups have been hardly sharing their knowledge. This is the main reason why we frequently find plant misidentifications in the papers written by non-botanists, while ‘pure’ botanists often fail to spell correctly, to trace the root of vernacular names and/or to report adequately the uses of the plants they study. Such ‘communication gaps’ may cause risky misidentifications due to the transfer of erroneous information concerning the alimentary use and therapeutic properties of some poisonous plants (i.e., Corsi and Pagni, 1979; Provitina, 1991; Atzei, 2003; Giardina et al., 2007; Łuczaj et al., 2012; see Supplementary Table S3).

The use of toxic and poisonous plants is a common pattern worldwide, especially were seasonality strongly affects food availability, and local human communities learned to exploit even the less attracting resources. This fact is not only a consequence of famine but also a question of timing and a result of century-old folk knowledge: in fact, some plants may contain a much lower concentration of poisonous compounds in some periods of the year and/or in some specific organs. Moreover, the fact that some communities use a species does not necessarily mean it is safe to eat it. Hence, detailed data on the gathering and cooking procedures are of paramount importance, and such information should be taken into account before discarding any poisonous plant from the list of edible ones: this is the case of several species which are cooked before being consumed as vegetables (like the tender shoots of *Asphodeline lutea*, *Carlina gummifera*, *Clematis vitalba*, and *Tamus communis*) or to prepare jams (such as the fruits of *Rubia peregrina*).

The data reported by Geraci et al. (2018) are based on interviews carried out throughout the main island of Sicily and only focused on the plants and plant portions consumed as vegetables, while c. 25% of our interviews were carried out on the circum-Sicilian islets and regarded the whole spectrum of NWFP, including 90 aromatic and fruit-bearing plant species. The same value of harvest frequency was assigned to 42 out of the 202 plants (20.8%) of the common pool, while for 49 taxa (24.2%) the evaluation was consistently different (2–4 points of the adopted scale). Most of the differences between the two assessments may depend on the way (total number, geographical distribution and scope) the interviews were carried out by the two research groups.

Our results point out the need of making further efforts in order to retrieve and homogenize the information already available in the gray ethnobotanical literature, mostly published in Italian on regional and local papers which are often hard to find and to consult. To overcome errors and to improve the quality and effectiveness of their researches, ethnobotanists, ethnologists, and all the skilled persons involved in wild plant harvesting should start a tighter collaboration and launch an ambitious multidisciplinary research program focused on Sicilian traditional environmental knowledge (TEK: Heckler, 2012) and edible plants’ use.

In order to avoid the transfer of erroneous information, in our opinion, the international scientific community should perform a more severe review of the data concerning the human diet. As a rule of thumb, papers concerning edible plants should be not only written but also reviewed (and eventually rejected) by skilled local botanists in order to avoid that wrong information is published on scientific journals and reaches (and eventually kills) uninformed readers.

### Useful, Yet at the Brink of Oblivion

Taking into account the worrying forecasts regarding food security for the forthcoming years due to climate warming and global change (Ford-Lloyd et al., 2011), during the last decades an increasing effort has been addressed on the detection and conservation of CWR (Valdés et al., 1997; Heywood et al., 2007; Maxted et al., 2010; Dempewolf et al., 2014). Regional and national inventories have been strongly encouraged, and seed banks are currently created and implemented (Maxted et al., 2007, 2012; Vincent et al., 2013). These efforts aim at preserving wild food plants, restoring old varieties, finding out new technologies to enhance their use and new solutions to manage modern agricultural systems (Hajjar and Hodgkin, 2007) more sustainably (Caneva et al., 2013). In this framework, we are convinced that in-depth ecological insights on NWFP may innovate and increase the spectrum of cultivated vegetables.

In the perspective of implementing sustainable agriculture under the current global change scenario, each of the 39 plants reported in Table 1, showing the most promising traits in terms of tolerance to several stress factors (water shortage, high temperatures, and edaphic constraints), could be cultivated for experimental purposes. Also, given that a considerable number of Sicilian NWFP can be considered CWR, they could be tested in *ad hoc* genetic improvement programs of traditional crops, like it has been already done for *Allium* (Odeny and Narina, 2011), *Asparagus* (Falavigna et al., 2008; Kanno and Yokoyama, 2011), *Beta* (McGrath et al., 2011), *Daucus* (Grzebelus et al., 2011; Iorizzo et al., 2013), *Lactuca* (Davey and Anthony, 2011), *Vicia* (Bryant and Hughes, 2011), and for many Brassicaceae (*Brassica*: Branca and Cartea, 2011; *Diplotaxis*: Pignone and Martínez-Laborde, 2011; *Eruca*: Pignone and Gómez-Campo, 2011) and Rosaceae (Fragaria: Hummer et al., 2011; *Malus*: Ignatov and Bodishevskaya, 2011; *Prunus*: Potter, 2011; *Pyrus*: Bell and Itai, 2011; *Rubus*: Graham and Woodhead, 2011). Moreover, further research should be focused on many species-rich and promising families and genera of NWFP (Table 2) whose members could be used in domestication programs aiming at developing new crops: i.e., new fragrances such as thymes (e.g., *Thymbra capitata* and *Thymus spinulosus*) and other Lamiaceae such as *Clinopodium nepeta*, *Lavandula stoechas*, and *Micromeria juliana*, new vegetables such as several wild species of *Asparagus* and wild rockets (e.g., *Diplotaxis* spp., *Erucastrum virgatum*).

Any future breeding activity concerning the most promising NWFP should start from a better understanding of their ecological traits, preferring those species that may provide additional ecosystem services and belong to genera that proved to be prone to a sensitive shift of above- and below-ground biomass allocation during the domestication process (Denison, 2012; Milla et al., 2015, 2017).

Despite their potential and prominent interest, especially for future generations, most of Sicilian NWFP seem doomed to oblivion. Due to ongoing social and economic changes in the Mediterranean area, the vast majority of the last custodians of ethnobotanical knowledge are more than 70 years old. As a consequence of their aging, not only the traditional knowledge but also entire cultural landscapes – shaped by men for millennia and providing the most suitable habitat for plenty of NWFP – is deemed to be lost within the next 10–20 years. We will lose most of the diversity of NWFP if we will stop eating them. Interviews to last living memories of the past cultural heritage (De Gregorio, 2008; Sottile and Genchi, 2010) are urgently needed before traditional knowledge fades forever (Quave and Saitta, 2016; Cucinotta and Pieroni, 2018), in addition to the collection and the conservation of Sicilian wild and cultivated germplasm (Hammer and Perrino, 1995; La Mantia and Pasta, 2005; Portis et al., 2005; Hammer and Laghetti, 2006; Schicchi et al., 2008; Forconi and Guidi, 2013; Marino et al., 2013).

## Conclusion

In this paper, we provide some preliminary clues about the ecology of all Sicilian NWFP, but knowledge on this topic needs to be improved, in order to detect any discrepancy between their real frequency, their altitudinal range, their distribution pattern and their local use as a food resource. To do that, we still need to analyze the data of all the published vegetation surveys carried out in Sicily and to cover the ethnobotanical knowledge gaps in some sectors of the island.

Tightly connected with the traditional agro-pastoral practices and landscapes, many NWFP are becoming rarer and rarer as a consequence of the ongoing processes of land-use change and abandonment. The only way to maintain both TEK and NWFP is to envisage concrete and shared measures aiming at promoting the self-sustainment of traditional agro-silvopastoral practices at the European, the national and the regional level.

Humans have shaped natural landscapes worldwide since prehistoric times. However, they often succeeded to modify ecosystem services and functioning without destroying them (Kareiva et al., 2007; Willis et al., 2007). Instead of trying to come back to nature, which may result a hard and even anachronistic target (Willis and Birks, 2006), we should try to inherit and replicate the past practices combining extensive and sustainable land use with the conservation of species diversity and ecosystem services (Plieninger et al., 2006; Guarino and Pignatti, 2010). In the end, TEK in general, and NWFP use in particular, issue from a wise and dynamic combination of silvopastoral and crop farming activities, and land use practices which allowed people and landscape to co-evolve and co-occur over millennia (Mercuri et al., 2019).

We should try to learn more about the best solutions adopted by mankind throughout history, focusing on the co-evolution between weeds, plant harvesters and farmers. At the same time, we should make more efforts in order to detect and value the TEK of the last Mediterranean bio-cultural refugia (Barthel et al., 2013).

## Data Availability Statement

All datasets generated for this study are included in the article/Supplementary Material.

## Author Contributions

SP, FC, and RG conceived and supervised the project. AL carried out interviews across the whole Sicilian territory and built up the revised checklist of Sicilian NWFP with the help of SP and RG. SP, CM, RG, FC, and AG analyzed and interpreted the data. SP, RG, AG, GG, CM, and FC wrote the first draft. All authors made a substantial, direct and intellectual contribution to this work. All authors approved the final version of the manuscript.

## Conflict of Interest

The authors declare that the research was conducted in the absence of any commercial or financial relationships that could be construed as a potential conflict of interest.
