# Supplementary figures and images for: An Updated Checklist of the Sicilian Native Edible Plants: Preserving the Traditional Ecological Knowledge of Century-Old Agro-Pastoral Landscapes
Source: Front Plant Sci. 2020 Apr 29;11:388. doi: 10.3389/fpls.2020.00388 (PMC7201097; doi:10.3389/fpls.2020.00388)

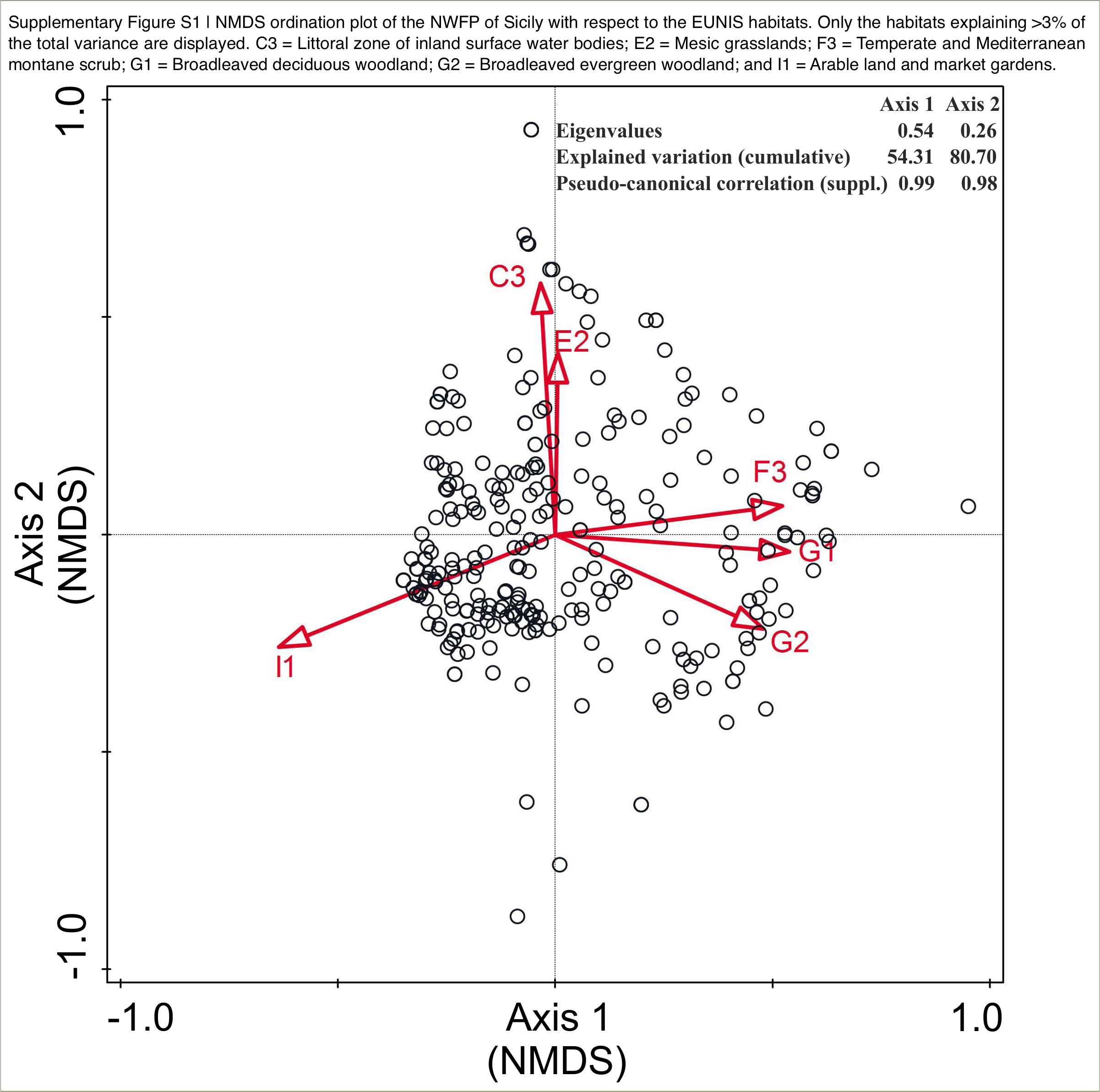

Supplement: Supplementary file 1 [file Image_1.tif]
