# Supplementary material for: An Updated Checklist of the Sicilian Native Edible Plants: Preserving the Traditional Ecological Knowledge of Century-Old Agro-Pastoral Landscapes
Source: Front Plant Sci. 2020 Apr 29;11:388. doi: 10.3389/fpls.2020.00388 (PMC7201097; doi:10.3389/fpls.2020.00388)
Supplement: Supplementary file 2 [file Table_1.PDF]

Supplementary Table S1. Critical and updated inventory of the Sicilian NWFP. The asterisks following plant names refer to plant taxa whose native status remains uncertain at the regional level.

Scientific name according to Pignatti et al. (2017-2019)	Synonyms adopted in the consulted literature	Family (APG IV, 2016)	Regional frequency of use as food plant (from Geraci et al., 2018)	Regional frequency of use as food plant (A. La Rosa, pers. data)	Edible parts	Use	Toxic (T) in large amounts or Poisonous (P) if eaten raw without previous treatment	Habit	Growth form	Minimum altitude (m)	Maximum altitude (m)	Ellenberg Indicator Values							CWR	Preferential Habitat - EUNIS	Preferential Habitat - Pignatti et al. (2017-19)
												L	T	C	U	R	N	S			
Achillea ligustica All.		Asteraceae	n.a.	1	le	R + C	T	perennial herb	scapose	0	800	7	8	4	3	6	2	0		E1, E5, G1, G2	EugBrecce, EugPrarid, SinCaorvi, SinIncurb
Aetheorhiza bulbosa (L.) Cass.	*Sonchus bulbosus* (L.) N.Kilian & Greuter subsp. *bulbosus*	Asteraceae	n.a.	1	le	R		geophyte	rosulate	0	600	7	8	4	3	5	3	0		B1, E1, I1	EugSand, SinCaorvi, SinIncurb
Alliaria petiolata (M.Bieb.) Cavara & Grande	*Alliaria officinalis* Andrz. ex DC.	Brassicaceae	2	1	bu, le, fr	R + C		perennial herb	scapose	0	1700	5	6	5	5	7	9	0		E5, F9, G1	EugBofor, EugRamarb, SinIncurb, SinMastaco
Allium ampeloprasum L.		Amaryllidaceae	5	3	bu, le, t-s	R + C		geophyte	scapose	0	1200	7	7	5	3	6	5	0	X	E1, G2, I1, J2	EugMagar, EugPrarid, EugRocce, EugRufasco, SinCaorvi, SinIncurb
Allium commutatum Guss.		Amaryllidaceae	n.a.	3	bu, le, t-s	R + C		geophyte	scapose	0	400	7	9	5	2	6	2	0	X	A2, B3, E1	EugMagar, EugPrarid, EugRocce, EugRufasco, EugSand
Allium nigrum L.		Amaryllidaceae	4	1	bu	R + C		geophyte	scapose	0	1000	10	9	4	3	6	4	0		E1, G2, I1	SinCaorvi, SinIncurb
Allium pendulinum Ten.		Amaryllidaceae	4	1	bu	R + C		geophyte	scapose	0	800	6	9	4	4	6	8	0		F9, G1, G2	EugBofor, EugGrefra, EugRamarb, EugRocce
Allium roseum L.		Amaryllidaceae	3	1	bu	R + C		geophyte	scapose	0	700	8	8	4	3	6	5	0		E1, F6, I1	EugMagar, EugPrarid, EugRocce, EugSand, SinCaorvi, SinIncurb
Allium subhirsutum L.		Amaryllidaceae	3	2	bu	R + C		geophyte	scapose	0	600	8	9	4	2	4	2	0		E1, F5, G1, G2	EugMagar, EugPrarid, EugRocce, SinCaorvi, SinIncurb, SinMuri
Allium triquetrum L.		Amaryllidaceae	3	3	bu	R + C		geophyte	caespitose	0	600	6	9	4	4	4	7	0		G1, G2	EugBofor, EugRamarb
Allium ursinum L.		Amaryllidaceae	2	1	bu, le	R + C		geophyte	scapose	0	1500	2	NA	5	6	7	8	0		G1	EugPrarid, SinCaorvi, SinIncurb
Ammi majus L.		Apiaceae	2	1	le	R + C	T	annual herb	scapose	0	1300	11	9	5	4	5	2	0		E6, I1	EugPrarid, SinCaorvi, SinIncurb
Anacamptis morio (L.) R.M.Bateman, Pridgeon & M.W. Chase	*Orchis morio* L., "*Orchis palustris*" *sensu* Pasta et al. (2011)	Orchidaceae	n.a.	1	bu	C		geophyte	scapose	0	1800	7	5	4	4	7	3	0		E1, E2, F5, F6	EugPrarid, SinIncurb
Anacyclus clavatus (Desf.) Pers.	*Anacyclus tomentosus* DC.	Asteraceae	2	1	t-s	C		annual herb	scapose	0	500	8	9	4	4	4	2	0		E1, E5, E6, I1, J2	SinCaorvi, SinIncurb
Anthemis arvensis L. *s.l.*	[incl. subsp. *incrassata* (Loisel.) Nyman]	Asteraceae	2	1	le	C		annual herb	scapose	0	1800	7	6	4	4	3	6	0		I1	EugBofor, EugRamarb
Anthriscus nemorosa (M.Bieb.) Spreng.		Apiaceae	n.a.	1	a-p	C	T	perennial herb	scapose	600	1870	5	4	6	5	5	5	0		E5, G1	AcqLapal, AcqPasal, EugPrapin, EugRamarb, SinCaorvi, SinIncurb
Apium graveolens L.*		Apiaceae	3	1	le, t-s	C	T	perennial herb	scapose	0	1500	7	7	5	7	5	7	0	X	C3	AcqToriflu, EugBrecce, EugGrefra, EugPrarid, EugRocce, EugRufasco
Arabis collina Ten.		Brassicaceae	2	1	t-s	C		perennial herb	caespitose	100	2000	7	5	3	4	8	3	0		H2, J2	EugBrecce, EugGrefra, EugPrarid, EugRocce, SinMuri
Arabis hirsuta (L.) Scop.		Brassicaceae	2	1	t-s	C		perennial herb	scapose	0	2000	7	5	5	4	8	NA	0		E1	EugPrarid, EugRamarb, EugRocce, SinCaorvi, SinIncurb, SinMuri
Arabis turrita L.	*Pseudoturritis turrita* (L.) Al-Shehbaz	Brassicaceae	2	1	le, t-s	C		perennial herb	scapose	400	1600	6	7	6	6	7	3	0		F5, G1	EugBofor, EugBrecce, EugPrarid, EugRamarb, EugRocce
Arbutus unedo L.		Ericaceae	n.a.	3	fr	R + C		tree	scapose	0	800	10	9	4	3	4	2	0		F5, F6, G1, G2	EugBofor, EugMagar
Arctium minus (Hill) Bernh.	"*Arctium lappa* L." *sensu Auct*.	Asteraceae	2	1	le, t-s	C		perennial herb	scapose	0	1500	10	5	5	5	8	9	0		E5	AcqToriflu, EugGrefra, EugRamarb, SinCaorvi, SinIncurb, SinMastaco
Asparagus acutifolius L.		Asparagaceae	5	5	t-s	C		scrub	caespitose	0	1300	6	9	4	2	5	5	0	X	F5, G1, G2	EugMagar, EugPrarid, EugRamarb, EugRocce, SinCaorvi, SinMuri
Asparagus albus L.		Asparagaceae	4	3	t-s	C		scrub	caespitose	0	1000	8	10	3	2	4	2	0	X	F5, F6	EugMagar, EugPrarid, EugRocce
Asparagus aphyllus L.		Asparagaceae	3	1	t-s	C		scrub	caespitose	0	900	11	11	4	2	5	2	0	X	F5, F6, G2	EugMagar, EugPrarid, EugRocce, EugRufasco
Asparagus horridus L.	*Asparagus stipularis* Forssk.	Asparagaceae	3	1	t-s	C		scrub	caespitose	0	500	11	11	4	2	5	2	0	X	A2, F5, F6	EugMagar, EugPrarid, EugRocce, EugRufasco
Asphodeline lutea (L.) Rchb.		Asphodelaceae	3	5	fl-b, t-s	C	P	geophyte	scapose	0	1700	11	6	6	2	6	3	0		E1, E5, F6	EugMagar, EugPrarid, EugRocce
Asphodelus ramosus L.	*Asphodelus microcarpus* Salzm. & Viv., *Asphodelus cerasifer* Gay	Asphodelaceae	2	1	bu	C	P	geophyte	rosulate	0	1200	10	9	4	2	3	5	0		E1, E5, F6	EugMagar, EugPrarid, EugRocce, SinIncurb
Astragalus boeticus L.		Fabaceae	n.a.	1	se	C		annual herb	scapose	0	50	11	11	5	1	3	1	0		E1, I1	EugPrarid, EugSand, SinIncurb
Barbarea bracteosa Guss.	"*Barbarea vulgaris* R.Br." *sensu Auct*.	Brassicaceae	n.a.	1	t-s	C		perennial herb	scapose	500	2000	7	6	5	7	5	5	0	X	C3, E5	AcqLapal, AcqToriflu, EugGrefra, EugPrapin, EugRamarb
Barlia robertiana (Loisel.) Greuter	*Himantoglossum robertianum* (Loisel.) P.Delforge	Orchidaceae	2	2	bu	C		geophyte	rosulate	0	1000	7	9	4	3	6	2	0		E1, F6, G2, I1	EugMagar, EugPrarid, SinIncurb
Bellis annua L. subsp. *annua*		Asteraceae	3	1	b-r	C		perennial herb	scapose	0	800	6	9	4	7	2	2	0		E1, E5, I1	AcqPasal, EugPrarid, EugRocce, SinCalp
Bellis perennis L. *s.l.*		Asteraceae	3	1	b-r	C		perennial herb	rosulate	0	1800	9	5	4	NA	NA	5	0		E1, E2, E5	AcqToriflu, EugPrapin, SinCalp, SinCaorvi, SinIncurb
Bellis sylvestris Cirillo		Asteraceae	2	1	b-r	C		perennial herb	rosulate	0	1400	5	8	4	3	3	3	0		E1, E5, G1, G3	EugRamarb, SinCaorvi, SinIncurb
Berula erecta (Huds.) Coville		Apiaceae	n.a.	1	a-p	C	P	perennial herb	scapose	0	1500	8	6	4	10	NA	7	0		C3	AcqLapal, AcqToriflu
Beta vulgaris L. subsp. *maritima* (L.) Arcang.		Amaranthaceae	5	5	le	C		annual herb	scapose	0	600	10	7	5	6	6	5	1	X	A2, I1, I2	AcqPasal, EugGrefra, SinCaorvi, SinIncurb
Biscutella maritima Ten.	"*Biscutella lyrata* L." *sensu Auct*.	Brassicaceae	2	1	t-s	C		annual herb	rosulate	0	1500	11	10	4	2	6	1	0		E1, I1	EugBrecce, EugGrefra, EugPrarid, EugRocce, SinCaorvi, SinIncurb
Borago officinalis L.		Boraginaceae	5	5	le, fl	C (le R)	T	perennial herb	caespitose	0	1400	7	8	5	3	5	5	0		E1, FB, G2, I1	EugGrefra, EugMagar, EugPrarid, SinCaorvi, SinIncurb
Brassica fruticulosa Cirillo		Brassicaceae	4	5	b-r, t-s, infl	C		scrub	caespitose	0	1200	8	8	4	4	4	4	0		E1, FB, I1, J2	EugBrecce, EugRocce, SinCaorvi, SinIncurb, SinMuri
Brassica incana Ten.		Brassicaceae	2	2	le, infl, t-s	R + C		scrub	scapose	0	800	8	10	3	2	7	1	0	X	H3	EugBrecce, EugMagar, EugRocce, EugRufasco
Brassica nigra (L.) Koch*		Brassicaceae	3	5	b-r, infl	C		perennial herb	scapose	0	800	8	7	5	4	4	4	0	X	I1	EugGrefra, EugPrapin, SinCaorvi, SinIncurb, SinMastaco
Brassica rapa L. subsp. *campestris* (L.) Clapham*		Brassicaceae	5	5	b-r, infl	C		annual herb	scapose	0	1000	8	7	5	4	4	4	0	X	E1, FB, G2, I1	EugGrefra, EugPrapin, SinCaorvi, SinIncurb, SinMastaco
Brassica rupestris Raf. *s.l.*		Brassicaceae	2	2	le, infl, t-s	R + C		scrub	scapose	200	1200	7	10	3	2	7	1	0	X	H3	EugBrecce, EugMagar, EugRocce, EugRufasco
Brassica tournefortii Gouan		Brassicaceae	2	2	le, t-s	R + C		perennial herb	caespitose	0	50	11	12	6	2	3	1	0		E1, FB, G2, I1	EugSand, SinCaorvi, SinIncurb
Bunias erucago L.		Brassicaceae	3	1	le	C		annual herb	scapose	0	2000	8	8	5	4	5	3	0		E1, FB, G2, I1	EugGrefra, EugPrarid, SinCaorvi, SinIncurb
Cakile maritima Scop.		Brassicaceae	2	1	le	C		annual herb	scapose	0	50	9	8	2	6	NA	8	3		A2	EugSand
Calendula arvensis (Vaill.) L.		Asteraceae	2	1	b-r, t-s, fl	R (fl) + C		annual herb	scapose	0	600	7	8	5	3	8	5	0		E1, FB, G2, I1	EugPrarid, SinCaorvi, SinIncurb
Capparis sicula Veill.		Capparaceae	4	4	fl-b, le, fr	S + C		scrub	caespitose	0	50	12	10	4	2	NA	1	3	X	H3, J2	EugMagar, EugRocce, SinIncurb
Capparis spinosa L. *s.l.*	[incl. subsp. *rupestris* (Sm.) Nyman]	Capparaceae	4	5	fl-b, le, fr, t-s	S + C		scrub	caespitose	0	1000	9	10	5	2	5	1	2	X	E6, F6	EugMagar, EugRocce, EugRufasco, SinCaorvi, SinIncurb
Capsella bursa-pastoris (L.) Medik.		Brassicaceae	2	1	b-r, le	C		annual herb	scapose	0	2000	7	NA	5	5	5	4	0		E1, FB, G2, I1, J2	EugGrefra, EugPrapin, EugRamarb, SinCalp, SinCaorvi, SinIncurb
Cardamine hirsuta L.		Brassicaceae	2	1	b-r, le	C		annual herb	scapose	0	1400	7	8	5	3	5	4	0		F3, F5, G2, I1, J2	AcqToriflu, EugBrecce, EugGrefra, EugRamarb, SinCaorvi, SinIncurb
Carduus argyroa Biv.		Asteraceae	2	2	t-s	C		annual herb	scapose	0	600	7	8	4	3	7	7	0		E5, J2	SinCaorvi, SinIncurb, SinMastaco
Carduus corymbosus Ten.		Asteraceae	2	1	t-s	C		annual herb	scapose	0	600	7	8	4	3	7	7	0		E5, J2	EugGrefra, EugMagar, EugPrarid, SinCaorvi, SinIncurb, SinMastaco
Carduus pycnocephalus L. *s.l.*		Asteraceae	2	2	le	C		annual herb	scapose	0	1250	7	8	4	3	NA	3	0		E1, E5, I1, J2	EugBrecce, EugGrefra, EugMagar, EugPrarid, EugRocce, SinMastaco
Carlina corymbosa L.		Asteraceae	n.a.	1	t-s	C		perennial herb	scapose	0	1200	6	NA	4	2	NA	4	0		E1, E5, F6, I1	EugBrecce, EugPrarid, SinIncurb
Carlina gummifera (L.) Less.	*Atractylis gummifera* L.	Asteraceae	2	2	infl	C	P	perennial herb	rosulate	0	700	11	11	5	1	7	1	0		E1, E5, F6	EugCalan, EugMagar, EugPrarid
Carlina hispanica Lam. subsp. *globosa* (Arcang.) Meusel & Kästner		Asteraceae	2	1	t-s	C		perennial herb	scapose	0	100	NA	NA	NA	NA	NA	NA	NA		E1, F6	EugBrecce, EugRocce, EugRufasco, SinIncurb
Carlina sicula Ten. subsp. *sicula*		Asteraceae	2	1	t-s	C		perennial herb	scapose	0	1600	11	11	3	1	2	1	0		E1, E5, F6	EugPrarid, SinCaorvi, SinIncurb
Carrichtera annua (L.) DC.		Brassicaceae	2	1	t-s	C		annual herb	scapose	0	300	11	11	4	2	4	2	0		E1, I1	EugMagar, EugPrarid, EugRocce, SinIncurb
Carthamus lanatus L.		Asteraceae	2	1	t-s	R		annual herb	scapose	0	1300	10	8	5	3	5	6	0		E1, E5, I1, J2	EugPrarid, SinCaorvi, SinIncurb
Carthamus pinnatus Desf.		Asteraceae	2	1	b-r	C		perennial herb	scapose	0	1400	11	8	3	3	6	2	0		E1, E5	EugMagar, EugPrarid, SinIncurb
Castanea sativa Mill.*		Fagaceae	n.a.	5	fr	R + C		tree	scapose	300	1200	5	8	6	NA	4	NA	0		G1	EugBofor
Celtis australis L.*		Cannabaceae	n.a.	3	fr	R		tree	scapose	0	800	7	8	5	3	7	4	0		F3, F5, G1, G2	EugBofor, EugBrecce, EugRamarb, EugRocce
Celtis tournefortii Lam. subsp. *aetnensis* (Tornab.) Raimondo & Schicchi		Cannabaceae	n.a.	2	fr	R		tree	scapose	500	800	6	8	3	3	4	2	0		F5, G2	EugBrecce, EugMagar, EugRocce, SinIncurb
Centaurea calcitrapa L.		Asteraceae	3	4	b-r	C		perennial herb	scapose	0	1400	10	9	5	3	NA	5	0		E5, I1, I2, J2	EugPrarid, SinCaorvi, SinIncurb
Centaurea napifolia L.		Asteraceae	3	2	b-r	C		annual herb	scapose	0	600	8	11	5	4	6	3	0		E5, FB, I1	EugPrarid, EugRamarb, SinCaorvi, SinIncurb
Centaurea sicula L.	*Centaurea nicaeensis* All.	Asteraceae	3	3	b-r, le	C		perennial herb	scapose	0	500	10	11	5	2	5	4	0		E1, E5, I1	SinCaorvi, SinIncurb

Supplementary Table S1. Critical and updated inventory of the Sicilian NWFP. The asterisks following plant names refer to plant taxa whose native status remains uncertain at the regional level.

Scientific name according to Pignatti et al. (2017-2019)	Synonyms adopted in the consulted literature	Family (APG IV, 2016)	Regional frequency of use as food plant (from Geraci et al., 2018)	Regional frequency of use as food plant (A. La Rosa, pers. data)	Edible parts	Use	Toxic (T) in large amounts or Poisonous (P) if eaten raw without previous treatment	Habit	Growth form	Minimum altitude (m)	Maximum altitude (m)	Ellenberg Indicator Values							CWR	Preferential Habitat - EUNIS	Preferential Habitat - Pignatti et al. (2017-19)
												L	T	C	U	R	N	S			
Centaurea solstitialis L. subsp. *schouwii* (DC.) Dostál		Asteraceae	3	3	le	C		perennial herb	scapose	0	1400	10	9	4	3	NA	5	0		E5, E6, J2	SinCaorvi, SinIncurb
Centaurium erythraea Rafn *s.l.*		Gentianaceae	n.a.	1	a-p	R	T	annual herb	scapose	0	1500	8	6	5	5	6	NA	0		C3, E1, F5, F6	EugCalan, EugGrefra, EugMagar, EugPrarid, EugRocce
Centranthus ruber (L.) DC		Valerianaceae	1	1	le	R + C		scrub	caespitose	0	1200	6	8	4	2	NA	1	0		H3, J2	EugBrecce, EugGrefra, EugMagar, EugRocce, EugRufasco, SinMuri
Ceratonia siliqua L.*		Fabaceae	n.a.	4	fr	R		tree	scapose	0	400	11	11	5	3	NA	3	0		F5	EugMagar, SinCaorvi
Cerinthe major L.	[incl. *Cerinthe aspera* Roth]	Boraginaceae	3	2	le, t-s, fl-n	R + C		annual herb	scapose	0	800	7	8	4	4	5	9	0		E1, FB, G2, I1	AcqToriflu, EugGrefra, EugPrapin, EugRamarb, SinCaorvi, SinIncurb
Chamaemelum fuscatum (Brot.) Vasc.	*Anthemis praecox* Link	Asteraceae	2	1	t-s	R + C		annual herb	scapose	0	600	8	8	4	3	3	2	0		E1, FB, I1	EugPrapin, EugSand, SinIncurb
Chamaerops humilis L.		Arecaceae	2	1	t-s	R		scrub	caespitose	0	600	11	10	3	1	4	1	0		F5, F6, G2	EugMagar, EugPrarid, EugRocce, EugRufasco
Chenopodiastrum murale (L.) S.Fuentes, Uotila & Borsch	*Chenopodium murale* L.	Amaranthaceae	n.a.	2	le, t-s	C		annual herb	scapose	0	900	8	7	5	4	NA	9	0		I2, J2	SinCaorvi, SinIncurb
Chenopodium album L.		Amaranthaceae	2	2	t-s	C		annual herb	scapose	0	2000	7	7	5	4	5	7	0		E5, I1, I2, J2	AcqToriflu, EugGrefra, SinCaorvi, SinIncurb
Chondrilla juncea L.		Asteraceae	3	1	b-r, le, t-s	R + C		perennial herb	scapose	0	1700	8	7	5	3	8	NA	0		E5, I1, I2, J2	EugPrarid, SinIncurb
Cichorium intybus L.*		Asteraceae	5	5	b-r, le	R + C		perennial herb	scapose	0	1450	9	6	5	3	8	5	0	X	E5, I1, I2	SinCaorvi, SinIncurb
Cichorium pumilum Jacq.		Asteraceae	5	2	b-r, le	R + C		perennial herb	scapose	0	100	NA	NA	NA	NA	NA	NA	NA	X	E2	SinCaorvi, SinIncurb
Clematis cirrhosa L.		Ranunculaceae	n.a.	1	le	C	P	liana	climbing	0	600	7	10	4	2	4	2	0		F5, G1, G2	EugMagar, EugRocce, SinIncurb
Clematis vitalba L.		Ranunculaceae	3	1	t-s	C	P	liana	climbing	0	1300	7	7	4	5	7	7	0		F3, G1, G2	AcqToriflu, EugBofor, EugRamarb, SinIncurb
Clinopodium nepeta (L.) Kuntze	*Calamintha nepeta* (L.) Savi	Lamiaceae	3	3	le, t-s	R + C		perennial herb	scapose	0	1500	5	7	5	3	9	3	0		E1, F6, I1	EugGrefra, EugPrapin, EugPrarid, EugRamarb, SinCaorvi, SinMastaco
Corylus avellana L.*		Betulaceae	n.a.	4	fr	R		scrub	caespitose	0	1700	6	5	4	5	5	8	0	X	F3, G1	EugBofor, EugRamarb, SinCaorvi
Crataegus monogyna Jacq.		Rosaceae	n.a.	2	fr	R + C		scrub	caespitose	0	1500	6	7	5	4	6	3	0	X	F5, G1, G2	EugBofor, EugRamarb
Crepis bursifolia L.		Asteraceae	3	2	b-r, le	C		perennial herb	rosulate	0	900	9	6	4	3	8	2	0		E1, I1, I2, J2	EugPrarid, SinIncurb
Crepis gussonei Greuter	*Crepis spathulata* Guss., *Crepis sprengelii* Nicotra	Asteraceae	2	1	b-r	C		perennial herb	rosulate	600	1200	8	10	4	3	NA	2	0		E1, G1, G2	EugRamarb, SinCaorvi, SinIncurb
Crepis leontodontoides All.		Asteraceae	3	1	b-r, le	C		perennial herb	rosulate	0	1800	5	8	4	4	3	7	0		E1, E5	EugRamarb, SinIncurb
Crepis neglecta L. subsp. *corymbosa* (Ten.) Nyman		Asteraceae	2	1	b-r	C		annual herb	scapose	0	800	7	6	3	4	6	3	0		E1, E5	EugBrecce, EugGrefra, SinCaorvi, SinIncurb
Crepis vesicaria L. *s.l.*	[incl. subsp. *hyemalis* (Biv.) Babc. and subsp. *taraxacifolia* (Thuill.) Thell.]	Asteraceae	4	4	b-r, le	C		annual herb	rosulate	0	1500	8	8	3	3	6	2	0		E1, E2, E5, I1	SinCaorvi, SinIncurb
Crithmum maritimum L.		Apiaceae	2	3	le, t-s, se	R		scrub	caespitose	0	50	11	8	2	1	NA	1	3		B3	EugBrecce, EugRufasco, SinIncurb, SinMuri
Crocus longiflorus Raf.		Iridaceae	n.a.	2	fl	R		geophyte	rosulate	0	1500	9	8	4	2	6	2	0	X	E1, E6	EugBofor, EugMagar, EugPrarid, EugRamarb, EugRocce
Cynara cardunculus L. *s.l.*	*Cynara horrida* Aiton	Asteraceae	4	5	t-s, fl-b	C		perennial herb	scapose	0	1200	10	9	4	5	6	3	0	X	E1, E5, E6	EugPrarid, SinCaorvi, SinIncurb
Daucus carota L. *s.l.*		Apiaceae	2	1	le, ro, b-r, t-s	R + C		annual herb	scapose	0	1400	8	6	5	4	5	4	0	X	E1, I1	AcqToriflu, EugGrefra, EugPrapin, EugPrarid, EugRamarb, SinCaorvi
Diplotaxis crassifolia (Raf.) DC.		Brassicaceae	3	2	le, t-s	C		perennial herb	caespitose	0	600	11	11	4	2	9	1	0	X	E1, E6, F6, H3	EugCalan, EugMagar, EugPrarid, EugRocce, EugRufasco
Diplotaxis erucoides (L.) DC.		Brassicaceae	4	4	le, t-s	R + C		annual herb	scapose	0	1400	8	8	4	3	5	5	0		FB, G2, I1	SinCaorvi, SinIncurb
Diplotaxis muralis (L.) DC.		Brassicaceae	2	1	le, t-s	C		annual herb	rosulate	0	1000	8	8	3	3	5	5	0		G2, H3, I1	EugGrefra, EugMagar, EugRocce, SinCaorvi, SinIncurb, SinMuri
Diplotaxis tenuifolia (L.) DC.		Brassicaceae	4	3	le, t-s	R + C		scrub	caespitose	0	1000	8	7	5	4	6	5	0	X	E1, FB, G2, I1, I2	EugGrefra, SinCaorvi, SinIncurb
Echinophora spinosa L.		Apiaceae	n.a.	1	le, ro	R	T	perennial herb	scapose	0	50	12	8	5	4	7	1	2		B1	EugSand
Echium italicum L. subsp. *siculum* (Lacaita) Greuter & Burdet		Boraginaceae	2	1	b-r, le	C		perennial herb	scapose	0	1300	10	8	5	3	3	4	0		E1, E5	EugGrefra, EugMagar, EugPrarid, EugRamarb, SinCaorvi, SinIncurb
Echium plantagineum L.		Boraginaceae	2	1	le	C		annual herb	caespitose	0	1300	10	8	5	3	5	5	0		E1, E5, I1	EugBrecce, EugGrefra, EugPrarid, EugRocce, EugSand, SinCaorvi
Echium vulgare L. subsp. *pustulatum* (Sm.) em. Schmid & Gams		Boraginaceae	n.a.	1	le, infl, t-s	C		perennial herb	scapose	0	1700	9	7	5	4	5	4	0		E1, E5	EugGrefra, EugPrarid, EugRamarb, SinCaorvi, SinIncurb
Equisetum telmateia Ehrh.		Equisetaceae	n.a.	1	a-p	C		geophyte	scapose	0	1700	5	7	4	8	8	5	0		E5, G1	AcqToriflu, EugRamarb, SinCaorvi, SinIncurb
Erodium moschatum (L.) L'Hér.		Geraniaceae	n.a.	1	a-p	C		annual herb	caespitose	0	1300	11	9	5	2	5	2	0		E1, E5, I1, I2	EugPrarid, SinCaorvi, SinIncurb
Eruca sativa Mill.*	*Eruca vesicaria* (L.) Cav.	Brassicaceae	3	2	le, t-s	R + C		annual herb	scapose	0	800	7	8	6	3	5	5	0	X	E1, I1	SinCaorvi, SinIncurb
Erucastrum virgatum C. Presl subsp. *virgatum*	*Sinapis virgata* C.Presl	Brassicaceae	2	1	le, t-s	C		scrub	scapose	0	600	8	7	4	3	7	2	0		B3, H2	EugGrefra, EugMagar, EugRocce, EugRufasco
Eryngium campestre L.		Apiaceae	2	1	le, t-s	R		perennial herb	scapose	0	1500	9	7	5	3	8	3	0		E1, E5, I1	EugPrarid, SinCaorvi, SinIncurb
Fedia graciliflora Fisch. & C.A.Mey.	"*Fedia cornucopiae* (L.) Gaertn." *sensu Auct.*	Valerianaceae	3	3	b-r, le	R		annual herb	scapose	0	1200	7	9	4	2	5	1	0		E1, FB, G2, I1	EugGrefra, EugMagar, EugRocce, SinCaorvi
Ficaria verna Huds. *s.l.*	Ranunculus ficaria L.	Ranunculaceae	n.a.	1	fl-b, le, bu	S + C	P	perennial herb	caespitose	0	1300	4	5	5	6	7	7	0		E1, E2, E5, I1	AcqToriflu, EugBofor, EugGrefra, EugPrapin, EugRamarb, SinCaorvi
Ficus carica L. *s.l.**	[incl. var. *caprificus* (Risso) Tschirch & Ravasini]	Moraceae	n.a.	5	fr	R		tree	scapose	0	800	7	8	6	NA	5	NA	0	X	F9, G1, H3, J2	EugGrefra, EugRamarb, EugRocce, EugRufasco, SinCaorvi, SinIncurb
Foeniculum vulgare Mill. *s.l.*	[incl. subsp. *piperitum* (Ucria) Bég.], *Foeniculum officinale* All.	Apiaceae	5	5	se, le, t-s	R (se) + C		perennial herb	scapose	0	1000	9	8	5	3	7	7	0	X	E1, I1, J2	EugGrefra, EugMagar, EugPrarid, EugRocce, SinCaorvi, SinIncurb
Fragaria vesca L.		Rosaceae	n.a.	3	fr	R		perennial herb	rosulate	0	2100	6	NA	4	4	NA	5	0	X	G1	EugBofor, EugRamarb, SinCaorvi
Fraxinus angustifolia Vahl subsp. *oxycarpa* (M.Bieb. ex Willd.) Franco & Rocha Afonso		Oleaceae	n.a.	1	st-j	R		tree	scapose	0	1000	4	8	6	7	7	8	0		F9, G1	EugBofor, SinIncurb
Fraxinus ornus L.		Oleaceae	n.a.	2	st-j	R		tree	scapose	0	1400	5	8	6	3	8	3	0		F3, F5, G1, G2	EugBofor, EugPrarid, EugRamarb, EugRocce
Galactites tomentosus Moench		Asteraceae	2	2	t-s	C		perennial herb	scapose	0	1300	8	8	4	3	NA	7	0		E1, E5, I1	EugPrarid, EugRamarb, SinCaorvi, SinIncurb
Geum urbanum L.		Rosaceae	n.a.	1	t-s	R + C		perennial herb	scapose	0	1600	4	5	5	5	6	7	0		G1	EugBofor, EugRamarb, SinIncurb
Gladiolus byzantinus Mill.		Iridaceae	2	1	fl-n	R	T	geophyte	scapose	0	600	11	9	4	3	5	2	0		E1, F5, F6, I1	EugMagar, EugPrarid, EugRocce, SinCaorvi, SinIncurb
Gladiolus italicus Mill.	*Gladiolus segetum* Ker Gawl.	Iridaceae	2	1	fl-n	R	T	geophyte	scapose	0	1300	9	9	5	3	5	3	0		I1	EugPrapin, EugPrarid, SinCaorvi, SinIncurb
Glebionis coronaria (L.) Spach	*Chrysanthemum coronarium* L.	Asteraceae	2	1	t-s, fl	R (fl) + C		annual herb	scapose	0	600	7	9	4	3	4	4	0		E1, I1, I2, J2	SinCaorvi, SinIncurb
Glycyrrhiza glabra L.*		Fabaceae	n.a.	2	ro	R		geophyte	scapose	0	1000	7	8	4	2	7	7	0	X	C3, E1, F9, I1	EugMagar, EugPrarid, EugRamarb, EugSand, SinCaorvi, SinIncurb
Hedypnois rhagadioloides (L.) F.W.Schmidt		Asteraceae	2	1	a-p	C		annual herb	rosulate	0	1000	11	9	4	3	7	2	0		E1, I1	EugMagar, EugPrarid, SinCaorvi, SinIncurb
Hedypnois cretica (L.) Dum.Cours.		Asteraceae	2	1	a-p	C		annual herb	rosulate	0	600	8	8	4	3	5	4	0		E1, I1	EugMagar, EugPrarid, SinCaorvi, SinIncurb
Helichrysum italicum (Roth) G.Don subsp. *siculum* (Jord. & Fourr.) Galbany, L.Sáez & Benedí		Asteraceae	n.a.	1	le	R		scrub	caespitose	0	1400	8	8	5	4	3	2	0		E1, F9, H2	EugBrecce, EugGrefra, EugMagar, EugRocce, EugRufasco
Helminthotheca echioides (L.) J.Holub	*Picris echioides* L., *Helminthia echioides* (L.) Gaertn.	Asteraceae	2	3	a-p	R + C		annual herb	scapose	0	1300	11	8	5	2	NA	2	0		E1, E5, G2, I1, I2	EugPrarid, EugRamarb, SinCaorvi, SinIncurb
Helosciadium nodiflorum (L.) W.D.J.Koch subsp. *nodiflorum*	*Apium nodiflorum* (L.) Lag.	Apiaceae	3	3	le, t-s	R + C	T	perennial herb	caespitose	0	1200	7	8	5	10	NA	6	0	X	C3	AcqLapal, AcqToriflu, EugGrefra
Hermodactylus tuberosus (L.) Mill.	*Iris tuberosa* L.	Iridaceae	3	2	bu	C		geophyte	scapose	0	1400	7	7	5	3	5	3	0		E1, E5, I1	EugBofor, EugMagar, EugRamarb, SinCaorvi
Hirschfeldia incana (L.) Lagr.-Foss.*	*Sinapis incana* L.	Brassicaceae	4	4	infl, le, t-s	C		annual herb	scapose	0	1100	9	9	5	3	3	2	0		E1, E5, I1	EugGrefra, EugPrarid, SinCaorvi, SinIncurb
Hyoseris radiata L.		Asteraceae	4	2	b-r, le	R + C		perennial herb	rosulate	0	1000	11	8	4	2	7	1	0		E1, F5, F6, H2, I1	EugRocce, EugRufasco
Hyoseris scabra L.		Asteraceae	2	1	a-p	C		annual herb	rosulate	0	400	11	9	3	1	7	1	0		E1	EugRocce, EugRufasco
Hypochaeris achyrophorus L.		Asteraceae	4	1	a-p	R		annual herb	scapose	0	1200	10	9	4	2	NA	2	0		E1, F6	EugPrarid, SinCaorvi, SinIncurb
Hypochaeris cretensis (L.) Chaub. & Bory		Asteraceae	3	1	b-r, le	R		perennial herb	scapose	0	1800	7	8	4	2	7	1	0		E1, G2, I1	EugBrecce, EugPrarid
Hypochaeris glabra L.		Asteraceae	4	1	a-p	C		annual herb	rosulate	0	1200	10	8	5	2	2	1	0		E1	EugPrarid, SinCaorvi, SinIncurb
Hypochaeris laevigata (L.) Ces., Pass. & Gibelli		Asteraceae	4	2	b-r, le	C		perennial herb	scapose	0	1700	6	8	3	2	7	1	0		E1, H3	EugBrecce, EugGrefra, EugRufasco
Hypochaeris radicata L.		Asteraceae	4	4	b-r, le	C		geophyte	rosulate	0	1500	9	8	4	2	NA	1	0		E1, E2, E5	EugPrarid, EugSand, SinCaorvi, SinIncurb
Juncus acutus L.		Juncaceae	2	1	t-s	C		perennial herb	caespitose	0	50	11	8	3	8	8	3	3		A2	AcqPasal, EugCalan, EugSand

Supplementary Table S1. Critical and updated inventory of the Sicilian NWFP. The asterisks following plant names refer to plant taxa whose native status remains uncertain at the regional level.

Scientific name according to Pignatti et al. (2017-2019)	Synonyms adopted in the consulted literature	Family (APG IV, 2016)	Regional frequency of use as food plant (from Geraci et al., 2018)	Regional frequency of use as food plant (A. La Rosa, pers. data)	Edible parts	Use	Toxic (T) in large amounts or Poisonous (P) if eaten raw without previous treatment	Habit	Growth form	Minimum altitude (m)	Maximum altitude (m)	Ellenberg Indicator Values							CWR	Preferential Habitat - EUNIS	Preferential Habitat - Pignatti et al. (2017-19)
												L	T	C	U	R	N	S			
Juniperus communis L.	[incl. subsp. *hemisphaerica* (J.Presl & C.Presl) Nyman]	Cupressaceae	n.a.	1	fr	R	T	scrub	caespitose	0	1500	8	NA	NA	4	NA	4	0		F7, G1, G3	EugPrarid, EugRamarb
Kundmannia sicula (L.) DC.		Apiaceae	1	2	b-r, le	C	T	perennial herb	scapose	0	1400	7	9	4	3	7	7	0		E1, F6	EugMagar, EugPrarid, SinCaorvi, SinIncurb
Lactuca muralis (L.) Gaertn.	*Mycelis muralis* (L.) Dumort.	Asteraceae	3	1	le	R + C		perennial herb	scapose	200	1800	4	5	4	5	NA	6	0		E5, G1, G2	EugBofor, EugRamarb
Lactuca serriola L.*	*Lactuca scariola* L.	Asteraceae	3	1	le	R + C		perennial herb	scapose	0	800	9	7	7	4	6	4	0	X	I1, I2, J2	SinCaorvi, SinIncurb
Lactuca viminea (L.) C.Presl		Asteraceae	3	1	b-r	R + C		perennial herb	scapose	0	1900	6	7	5	3	7	3	0		E2, E5, H2	EugBrecce, EugGrefra, EugPrarid, SinCaorvi, SinIncurb
Lamium flexuosum Ten.		Lamiaceae	2	1	fl-n, st-j	R		perennial herb	scapose	300	1900	5	5	4	6	7	7	0		E5, G1, G2	EugBofor, EugGrefra, EugRamarb, EugRocce, SinMuri
Lathyrus annuus L.		Fabaceae	2	1	t-s	C		annual herb	climbing	0	1500	8	8	5	3	5	2	0		E1, I1	EugMagar, EugPrarid, SinCaorvi
Lathyrus articulatus L.		Fabaceae	n.a.	1	se	R + C		annual herb	climbing	0	600	11	9	4	2	5	2	0		E1, I1	EugMagar, EugPrarid, SinCaorvi
Lathyrus cicera L.		Fabaceae	n.a.	1	se	R + C		annual herb	climbing	0	1000	8	8	5	3	5	2	0	X	E1, I1	EugMagar, EugPrarid, SinCaorvi
Lathyrus clymenum L.		Fabaceae	n.a.	1	t-s, se	C		annual herb	climbing	0	1100	7	8	4	4	3	3	0		E1, I1	EugMagar, EugPrarid, SinCaorvi, SinIncurb
Lathyrus ochrus (L.) DC.		Fabaceae	n.a.	1	se	C		annual herb	climbing	0	1000	7	8	4	2	5	2	0		E1, I1	EugMagar, EugPrarid, SinCaorvi
Lathyrus odoratus L.		Fabaceae	n.a.	1	se	C	T	annual herb	climbing	0	600	7	7	4	4	5	5	0		E1, E2, E5, F3	EugCalan, EugMagar, EugPrarid, SinCaorvi, SinIncurb
Lathyrus sylvestris L.		Fabaceae	2	2	infl, t-s, fl-b	C	P (se)	perennial herb	climbing	0	1500	7	5	6	4	4	4	0		E1, E2, E5, F3	EugGrefra, EugPrarid, EugRamarb, SinIncurb
Laurus nobilis L.		Lauraceae	n.a.	5	le	C		tree	scapose	0	800	2	7	4	8	4	6	0	X	F3, F5, G1, G2	EugBofor, EugRamarb
Lavandula stoechas L.		Lamiaceae	n.a.	2	fl	R		scrub	caespitose	0	600	11	9	4	2	1	1	0		F5, F6, G2	EugMagar
Lavatera trimestris L.		Malvaceae	3	1	le	C		annual herb	caespitose	0	800	8	9	4	2	5	4	0		E1, I1	EugMagar, SinCaorvi, SinIncurb
Leontodon siculus (Guss.) Nyman		Asteraceae	3	1	b-r	C		geophyte	rosulate	900	1800	NA	NA	NA	NA	NA	NA	NA		E1, E2, E5	EugBofor, EugPrapin
Leontodon tuberosus L.	*Thrincia tuberosa* (L.) DC.	Asteraceae	3	1	b-r	C		geophyte	rosulate	0	1000	11	8	4	3	7	2	0		E1, E5, I1	EugPrarid, EugRamarb, SinCaorvi
Lepidium draba L.	*Cardaria draba* (L.) Desv.	Brassicaceae	2	1	b-r, t-s	C		perennial herb	scapose	0	1500	8	7	7	3	8	4	0		E2, E5	EugMagar, EugPrarid, SinCaorvi, SinIncurb
Lepidium graminifolium L.		Brassicaceae	2	1	b-r, t-s	C		annual herb	scapose	0	600	8	8	5	3	NA	3	0		E5, I1, I2, J2	SinCaorvi, SinIncurb
Lepidium latifolium L.*		Brassicaceae	2	1	b-r, t-s	C		annual herb	scapose	0	1000	9	7	7	3	7	2	1		C3, D5, E2, I2	AcqPasal, EugSand, SinCaorvi, SinIncurb
Lobularia maritima (L.) Desv.	*Alyssum maritimum* (L.) Lam.	Brassicaceae	2	1	t-s, le	R + C		perennial herb	caespitose	0	1200	8	9	4	2	NA	1	0		E1, H2, I1	EugBrecce, EugGrefra, EugPrarid, EugRocce, SinCaorvi, SinIncurb
Lotus biflorus Desr.	"*Tetragonolobus conjugatus* (L.) Link" *sensu* Pasta et al. (2011)	Fabaceae	n.a.	1	fr	R		annual herb	caespitose	0	800	7	8	5	3	3	3	0		E1, I1	AcqFapost, EugPrapin, EugPrarid, EugRamarb, SinCaorvi
Lotus cytisoides L.		Fabaceae	n.a.	1	fr	C		scrub	caespitose	0	50	11	10	3	1	NA	1	2		B3, E1, H2	EugBrecce, EugRocce, EugRufasco, EugSand
Lotus edulis L.		Fabaceae	n.a.	1	fr	R		annual herb	caespitose	0	900	9	8	4	2	5	3	0		E1, I1	EugGrefra, EugMagar, EugPrarid, EugRocce, SinIncurb
Lotus tetragonolobus L.	*Tetragonolobus purpureus* Moench	Fabaceae	n.a.	1	fr	R		annual herb	scapose	0	1200	8	6	4	6	9	6	0		E1, I1	EugCalan, EugMagar, EugPrarid, EugRamarb, SinCaorvi
Lycium europaeum L.*		Solanaceae	3	2	t-s	C	T	scrub	caespitose	0	800	11	7	5	3	5	2	0		F3, F5, F6	EugGrefra, EugMagar, EugSand, SinIncurb
Lythrum salicaria L.		Lythraceae	n.a.	1	t-s	R + C		perennial herb	scapose	0	1500	7	5	5	8	7	NA	0		C3, D5, E2	AcqLapal, AcqToriflu, EugGrefra
Malva cretica Cav.		Malvaceae	3	1	le	C		annual herb	scapose	0	1300	7	7	4	4	7	8	0		E1, I1, J2	EugCalan, EugMagar, EugPrarid, SinCaorvi, SinIncurb
Malva multiflora (Cav.) Soldano, Banfi & Galasso	*Lavatera cretica* L.	Malvaceae	3	1	le	C		scrub	scapose	0	600	8	9	4	2	5	4	0		E5, I2	SinCaorvi, SinIncurb
Malva nicaeensis All.		Malvaceae	3	1	le	C + R		perennial herb	scapose	0	800	7	8	4	4	7	8	0		I1, I2, J2	EugPrarid, SinCaorvi, SinIncurb, SinMastaco
Malva parviflora L.		Malvaceae	3	1	le	C		perennial herb	scapose	0	800	7	8	5	4	7	8	0		I1, I2, J2	EugPrarid, SinCaorvi, SinIncurb, SinMastaco
Malva sylvestris L. *s.l.*		Malvaceae	3	1	le, t-s, se	C + R (se)		perennial herb	scapose	0	1600	8	6	4	4	NA	8	0		I1, I2, J2	EugRamarb, SinCalp, SinCaorvi, SinIncurb, SinMastaco
Matricaria chamomilla L.	*Chamomilla recutita* (L.) Rauschert	Asteraceae	n.a.	1	a-p	C + R		annual herb	scapose	0	1500	7	5	5	6	5	5	0	X	I1	SinCaorvi, SinIncurb
Melissa officinalis L. subsp. *altissima* (Sm.) Arcang.		Lamiaceae	n.a.	1	le	R		perennial herb	scapose	0	1000	6	7	5	4	6	4	0		E5, F9, G1	EugRamarb, SinCaorvi, SinIncurb
Mentha aquatica L.		Lamiaceae	n.a.	1	le	R		geophyte	scapose	0	1200	7	5	5	9	7	4	0		C3, D5, E2	AcqLapal, AcqToriflu, EugPrapin, SinIncurb
Mentha pulegium L.		Lamiaceae	n.a.	1	le	R		perennial herb	caespitose	0	1200	8	7	5	7	NA	2	0		C3, E2	AcqFapost, AcqLapal, AcqToriflu, EugGrefra, EugPrapin, SinCaorvi
Mentha spicata L.	*Mentha viridis* (L.) L.	Lamiaceae	n.a.	1	le	R		geophyte	scapose	0	1200	7	6	5	8	8	6	0	X	C3, D5, E2	AcqFapost, AcqLapal, AcqToriflu, EugPrapin, SinCaorvi, SinIncurb
Mentha suaveolens Ehrh.		Lamiaceae	n.a.	1	le	R		geophyte	scapose	0	1500	7	8	5	8	7	6	0	X	C3, D5, E2	AcqFapost, AcqLapal, AcqToriflu, EugPrapin, SinCaorvi, SinIncurb
Mespilus germanica L.		Rosaceae	n.a.	2	fr	R		Tree	scapose	0	1000	5	8	6	4	NA	NA	0		G2	EugBofor, EugRamarb, SinCaorvi
Micromeria juliana (L.) Benth. ex Rchb.		Lamiaceae	n.a.	1	fl	R		scrub	caespitose	0	1950	8	8	4	2	1	1	0		E6, H3	EugBrecce, EugMagar, EugRocce, EugRufasco, SinMuri
Microthlaspi perfoliatum (L.) F.K.Mey.	*Thlaspi perfoliatum* L.	Brassicaceae	2	1	a-p	C		annual herb	scapose	0	1950	8	6	5	4	8	2	0		I1	AcqStisori, AcqToriflu, EugPrapin, EugRamarb, SinCaorvi, SinIncurb
Moricandia arvensis (L.) DC.		Brassicaceae	2	1	a-p	C		annual herb	scapose	0	600	8	8	5	2	5	2	0		A2, E6, F6, H2	EugCalan, EugPrarid, EugRocce, SinIncurb
Muscari comosum (L.) Mill.	*Leopoldia comosa* (L.) Parl.	Asparagaceae	n.a.	1	bu	C	P	geophyte	rosulate	0	1500	7	8	5	3	7	NA	0		I1	EugMagar, EugPrarid, EugRocce, SinCaorvi
Myrtus communis L.		Myrtaceae	n.a.	3	fr, le, fl	C + R (fr, fl)	T	scrub	caespitose	0	500	8	9	4	3	5	2	0		F5, G2	EugMagar
Narcissus tazetta L. *s.l.*		Amaryllidaceae	1	1	infl, fl-b	C	P	geophyte	rosulate	0	1400	8	8	4	4	5	4	0		E1, E2, E5	EugMagar, EugPrapin, EugPrarid, EugRamarb, EugRocce, SinCaorvi
Nasturtium officinale R.Br.		Brassicaceae	3	2	le, t-s	C + R		perennial herb	caespitose	0	1800	7	4	5	11	7	7	0	X	C3	AcqLapal, AcqStisori, AcqToriflu, EugGrefra
Notobasis syriaca (L.) Cass.		Asteraceae	2	3	t-s	C + R		annual herb	scapose	0	1100	11	10	4	2	7	7	0		E5, J2	EugGrefra, EugPrarid, SinCaorvi, SinIncurb, SinMastaco
Oenanthe pimpinelloides L.		Apiaceae	n.a.	1	a-p	C		perennial herb	scapose	0	800	5	7	3	4	5	4	0		E2, E5, G1	AcqStisori, EugBofor, EugRamarb
Olea europaea L. var. *sylvestris* (Mill.) Lehr.		Oleaceae	n.a.	1	fr	R		tree	scapose	0	900	11	10	4	1	NA	2	0	X	F5, G1, G2	EugMagar, EugRocce, SinCaorvi
Onopordum horridum Viv.		Asteraceae	4	1	b-r, t-s	C		perennial herb	scapose	0	1500	11	6	4	3	7	9	0		E5	EugPrarid, EugRamarb, SinCaorvi, SinIncurb, SinMastaco
Onopordum illyricum L.		Asteraceae	4	3	b-r, t-s	C		perennial herb	scapose	0	1200	11	8	4	3	7	9	0		E5	EugPrarid, EugRamarb, SinCaorvi, SinIncurb, SinMastaco
Origanum vulgare L. subsp. *viridulum* (Martrin-Donos) Nyman	*Origanum heracleoticum* L., "*Origanum vulgare* L." *sensu* Auct.	Lamiaceae	n.a.	5	le	R		scrub	scapose	0	1700	7	6	5	3	NA	3	0	X	E1, E5, G1, G2	EugPrarid, EugRamarb, EugRocce
Orobanche crenata Forssk.		Orobanchaceae	n.a.	1	a-p	n.a.		annual herb	scapose	0	1400	8	5	6	3	5	4	0		B1, E1, E2, F6, I1	EugMagar, EugRamarb, SinCaorvi, SinIncurb
Oxalis corniculata L.		Oxalidaceae	n.a.	1	st-j	R	T	annual herb	creeping	0	800	7	7	NA	4	NA	6	0		J2	SinCalp, SinCaorvi, SinIncurb, SinMuri
Papaver rhoeas L.*		Papaveraceae	3	1	le, t-s	C + R		annual herb	scapose	0	1950	6	6	5	5	7	NA	0		G2, I1, I2	SinCaorvi, SinIncurb
Papaver setigerum DC.*		Papaveraceae	1	1	le	C + R		annual herb	scapose	0	600	11	9	4	2	7	1	0		I1	EugGrefra, EugRamarb, SinIncurb
Parietaria judaica L.	*Parietaria diffusa* Mert. & W.D.J.Koch, "Parietaria officinalis L." *sensu Auct.*	Urticaceae	n.a.	1	le	C		perennial herb	caespitose	0	1000	7	8	5	3	NA	6	0		G2, H3, I2, J2	EugMagar, EugRocce, SinIncurb, SinMuri
Phagnalon saxatile (L.) Cass.		Asteraceae	n.a.	1	le	R		scrub	caespitose	0	600	7	9	4	2	NA	1	0		E1, F6, H3, I1, J2	EugPrarid, EugRocce, EugRufasco, SinMuri
Phlomis fruticosa L.		Lamiaceae	n.a.	1	le	C + R		scrub	caespitose	0	500	11	9	5	3	7	2	0		F5, F6, H2	EugBrecce, EugMagar, EugRocce, EugRufasco
Picris hieracioides L.	[incl. subsp. *spinulosa* (Guss.) Arcang.]	Asteraceae	2	2	le	C		perennial herb	scapose	0	2000	8	NA	5	4	8	4	0		E1, E2, E5, I1	EugPrarid, SinIncurb
Pinus pinea L.		Pinaceae	n.a.	3	se	R		tree	scapose	0	800	11	8	5	2	4	3	0		F5, F6	EugBofor, EugMagar, EugSand, SinCaorvi
Pisum sativum L. subsp. *biflorum* (Raf.) Soldano	*Pisum sativum* L. subsp. *elatius* (M.Bieb.) Asch. & Gr.	Fabaceae	n.a.	2	se, fl	C + R		annual herb	climbing	0	1600	9	9	4	3	4	3	0		E1, E2, E5, F3	EugMagar, SinCaorvi, SinIncurb
Plantago afra L. *s.l.*	*Plantago psyllium* L.	Plantaginaceae	2	1	b-r	C		annual herb	scapose	0	900	11	6	4	3	7	2	0		E1, I1	EugGrefra, EugMagar, EugPrarid, EugSand, SinCaorvi, SinIncurb
Plantago coronopus L.		Plantaginaceae	3	1	b-r	C		perennial herb	rosulate	0	800	8	7	5	7	7	4	0		A2, E1, E2, E6, J2	AcqFapost, EugCalan, EugGrefra, EugPrarid, EugSand, SinCaorvi
Plantago lagopus L.		Plantaginaceae	2	1	b-r	C		annual herb	rosulate	0	1100	11	9	4	3	3	1	0		E1, E5, I1, I2, J2	EugCalan, EugMagar, EugPrarid, EugRocce, SinCaorvi, SinIncurb
Plantago lanceolata L.		Plantaginaceae	3	1	le	C		perennial herb	rosulate	0	1800	6	7	5	NA	NA	NA	0		E1, E2	EugCalan, EugGrefra, EugPrapin, EugPrarid, EugRamarb, EugRocce
Plantago major L.		Plantaginaceae	2	1	le	C		perennial herb	caespitose	0	1800	8	NA	NA	5	NA	7	0		E2, E5, I2	EugCalan, EugGrefra, EugPrapin, EugRamarb, SinCaorvi, SinIncurb

Supplementary Table S1. Critical and updated inventory of the Sicilian NWFP. The asterisks following plant names refer to plant taxa whose native status remains uncertain at the regional level.

Scientific name according to Pignatti et al. (2017-2019)	Synonyms adopted in the consulted literature	Family (APG IV, 2016)	Regional frequency of use as food plant (from Geraci et al., 2018)	Regional frequency of use as food plant (A. La Rosa, pers. data)	Edible parts	Use	Toxic (T) in large amounts or Poisonous (P) if eaten raw without previous treatment	Habit	Growth form	Minimum altitude (m)	Maximum altitude (m)	Ellenberg Indicator Values							CWR	Preferential Habitat - EUNIS	Preferential Habitat - Pignatti et al. (2017-19)
												L	T	C	U	R	N	S			
Plantago serraria L.		Plantaginaceae	3	1	le	C		perennial herb	rosulate	0	800	11	10	4	2	7	1	0		E1, F5, I1, I2, J2	AcqPasal, EugCalan, EugGrefra, EugPrarid, SinCaorvi, SinIncurb
Podospermum canum C.A.Mey.	*Scorzonera cana* (C.A.Mey.) Griseb.	Asteraceae	2	1	le, t-s	C		perennial herb	scapose	0	1200	9	8	6	5	9	4	1		A2, E6	EugCalan, EugPrarid, SinCaorvi, SinIncurb
Podospermum laciniatum (L.) DC.	*Scorzonera laciniata* L. [incl. subsp. *decumbens* (Guss.) Gemeinholzer & Greuter = *Podospermum calcitrapifolium* (Vahl) DC.]	Asteraceae	2	1	le, t-s	C + R		perennial herb	scapose	0	1900	7	8	6	3	8	NA	1		E6	EugCalan, EugPrarid, SinCaorvi, SinIncurb,
Portulaca oleracea L. *s.l.**		Portulacaceae	4	5	le, t-s	C + R		annual herb	caespitose	0	1700	7	8	5	4	7	7	0		G2, I1	EugRocce, SinCaorvi, SinIncurb, SinMuri
Primula vulgaris Huds.	*Primula acaulis* (L.) Hill	Primulaceae	2	1	b-r	C + R		perennial herb	rosulate	0	1800	6	5	4	5	7	5	0		G1, G2	EugBofor, EugPrapin, EugRamarb, SinCaorvi, SinIncurb
Prunus cupaniana Guss.		Rosaceae	n.a.	1	fr	R		scrub	caespitose	1250	1900	NA	NA	NA	NA	NA	NA	NA		F3, F7	EugMagar, EugRamarb, EugRocce, EugRufasco
Prunus mahaleb L.		Rosaceae	n.a.	1	fr	R		tree	scapose	0	1900	7	5	6	3	8	2	0		F3, G1	EugMagar, EugRamarb, EugRocce, EugRufasco
Prunus spinosa L.		Rosaceae	n.a.	3	fr	R		scrub	caespitose	0	1500	7	5	5	NA	NA	NA	0	X	F3, G1, G2	EugBofor, EugRamarb
Pyrus spinosa Forssk.		Rosaceae	n.a.	1	fr	R		tree	scapose	0	1100	7	8	4	4	7	3	0	X	F3, G1, G2	EugBofor, EugMagar, EugRamarb
Raphanus raphanistrum L. *s.l.**	[incl. subsp. *maritimus* (Sm.) Thell.]	Brassicaceae	4	4	a-p	C + R		annual herb	caespitose	0	1300	11	5	5	NA	4	5	0	X	A2, I1, I2	EugGrefra, SinCaorvi, SinIncurb
Rapistrum rugosum (L.) All.*	[incl. subsp. *orientale* (L.) Arcang. and *Rapistrum linnaeanum* Boiss. & Reut.]	Brassicaceae	4	1	a-p	C		annual herb	scapose	0	1100	7	7	5	4	5	5	0		I1	EugGrefra, EugPrarid, SinCaorvi, SinIncurb
Reichardia picroides (L.) Roth	*Picridium vulgare* Desf.	Asteraceae	5	4	b-r	C + R		perennial herb	rosulate	0	1000	7	8	4	3	6	2	0		B3, E1, I1, J2	EugPrarid, SinCaorvi, SinIncurb, SinMuri
Reseda alba L. *s.l.*		Resedaceae	n.a.	1	b-r, t-s	n.a.		annual herb	scapose	0	1000	11	8	4	3	7	1	0		E1, E5, I1, I2, J2	EugBrecce, EugGrefra, EugMagar, EugPrarid, SinIncurb
Rhagadiolus stellatus (L.) Gaertn.		Asteraceae	3	1	a-p	C		annual herb	scapose	0	1000	8	9	5	3	7	2	0		E1, I1	EugPrarid, SinCaorvi, SinIncurb
Ridolfia segetum (Guss.) Moris		Apiaceae	2	1	a-p	R	T	annual herb	scapose	0	1000	11	9	4	3	5	3	0		I1	SinCaorvi
Rorippa sylvestris (L.) Besser		Brassicaceae	1	1	le	C		perennial herb	scapose	0	1650	6	6	5	8	8	6	0		C3, E2, E5	AcqToriflu, EugGrefra, EugPrapin, SinCaorvi, SinIncurb
Rosa canina L.		Rosaceae	2	3	ps-fr, fl	C + R		scrub	caespitose	0	1500	8	5	5	4	NA	NA	0		F3	EugArbalp, EugBofor, EugBrecce, EugRamarb
Rosa sempervirens L.		Rosaceae	2	1	fl	C + R		liana	climbing	0	400	6	8	4	3	4	6	0		F3, F5, G1, G2	AcqToriflu, EugBofor, EugMagar, EugRamarb
Rosmarinus officinalis L.		Lamiaceae	n.a.	5	le	R		scrub	caespitose	0	800	10	8	4	2	6	1	0	X	F5, F6	EugBrecce, EugMagar, EugRocce, EugRufasco, SinIncurb
Rubia peregrina L.		Rubiaceae	n.a.	1	fr	C	P	scrub	climbing	0	1000	5	9	4	4	5	3	0		F3, F5, G1, G2	EugGrefra, EugPrarid
Rubus idaeus L.		Rosaceae	n.a.	1	fr, t-s	C + R		scrub	caespitose	100	1800	7	4	NA	5	5	8	0	X	F3	EugRamarb
Rubus ulmifolius Schott	"*Rubus fruticosus* L." *sensu Auct.*	Rosaceae	1	5	fr, t-s	C + R		scrub	caespitose	0	1100	5	8	5	4	5	8	0	X	F3, F5, G1, G2	EugRamarb, SinCaorvi, SinIncurb
Rumex bucephalophorus L.		Polygonaceae	3	1	a-p	C	T	annual herb	caespitose	0	1700	8	12	5	2	2	1	0		E1, FB, G2, I1	EugGrefra, EugPrarid, EugSand
Rumex crispus L.		Polygonaceae	1	1	le, t-s	C	T	perennial herb	scapose	0	1500	7	5	5	6	NA	5	0		C3, D5, E2	AcqToriflu, EugGrefra, EugPrapin, EugRamarb, SinCaorvi, SinMastaco
Rumex intermedius DC.		Polygonaceae	2	1	t-s	C	T	perennial herb	scapose	0	700	8	7	5	3	7	5	0		C3, E2, I1	SinCaorvi, SinIncurb
Rumex pulcher L. *s.l.*		Polygonaceae	2	1	a-p	C	T	annual herb	scapose	0	1750	8	8	5	2	6	9	0		E2, E5, I1	AcqToriflu, EugGrefra, EugRamarb, SinCaorvi, SinIncurb
Rumex scutatus L. *s.l.*	[incl. subsp. *aetnensis* (C.Presl) Cif. & Giacom. and subsp. *glaucescens* (Guss.) Brullo, Scelsi & Spamp.]	Polygonaceae	2	1	le, t-s, st-j	C + R	T	scrub	caespitose	50	3000	8	NA	6	4	7	3	0		H2	EugBrecce, EugGrefra, EugRocce
Rumex thyrsoides Desf.		Polygonaceae	2	1	le, t-s	C	T	perennial herb	scapose	0	1200	8	8	4	3	7	5	0		E1, E5, G2	EugMagar, EugPrarid, SinIncurb
Ruscus aculeatus L.		Asparagaceae	5	4	bu, t-s, se	C + R		scrub	scapose	0	1200	4	8	5	4	5	5	0		F3, G1, G2	EugBofor, EugMagar, EugRamarb
Salvia fruticosa Mill. subsp. *thomasii* (Lacaita) Brullo, Guglielmo, Pavone & Terrasi	*Salvia triloba* L.f.	Lamiaceae	n.a.	2	le	C + R		scrub	caespitose	0	1000	6	8	5	3	7	2	0	X	F5, F6	EugMagar, EugRocce, SinIncurb
Salvia sclarea L.		Lamiaceae	n.a.	2	le	R		perennial herb	scapose	0	900	5	8	5	3	7	2	0	X	E5	EugMagar, EugPrarid, EugRocce, SinIncurb
Sambucus nigra L.		Adoxaceae	2	3	fl, fr	C + R		tree	scapose	0	1400	7	5	4	5	NA	9	0		F3, G1	AcqToriflu, EugBofor, EugRamarb, SinMastaco
Sanguisorba minor Scop.		Rosaceae	1	2	le	C		perennial herb	scapose	0	1800	7	6	5	3	8	2	0		E1, F5, F6, G2, H3	EugBrecce, EugPrarid, EugRamarb, EugRocce
Scolymus grandiflorus Desf.		Asteraceae	4	3	le, t-s	C + R		geophyte	scapose	0	1400	11	10	3	2	5	3	0		E5	EugPrarid, SinCaorvi, SinIncurb
Scolymus hispanicus L.		Asteraceae	3	2	t-s	C + R		perennial herb	scapose	0	800	11	8	5	3	NA	2	0		B1, E5, I1	EugPrarid, EugSand, SinCaorvi, SinIncurb
Scolymus maculatus L.		Asteraceae	3	2	t-s	C + R		annual herb	scapose	0	600	11	11	5	2	5	3	0		E5	EugPrarid, SinCaorvi, SinIncurb
Scorzonera undulata Vahl subsp. *deliciosa* (DC.) Maire		Asteraceae	2	1	b-r, bu	C + R		geophyte	scapose	0	1000	8	8	5	3	6	2	0	X	E1, E6	EugPrarid, SinCaorvi, SinIncurb
Scorzoneroides cichoracea (Ten.) Greuter	*Leontodon cichoraceus* (Ten.) Sanguin.	Asteraceae	4	1	b-r	C		perennial herb	rosulate	150	1700	9	6	5	3	7	2	0		E1, E2, E5	EugPrarid, EugRamarb, SinIncurb
Scorzoneroides muelleri (Sch. Bip.) Greuter & Talavera subsp. *muelleri*	*Leontodon muelleri* (Sch.Bip.) Fiori	Asteraceae	4	1	b-r	C		annual herb	rosulate	0	800	11	11	5	3	7	2	0		E5, E6	EugPrapin, SinIncurb
Silene vulgaris (Moench) Garcke *s.l.*	[incl. subsp. *tenoreana* (Colla Soldano & Conti and subsp. *commutata* (Guss.) Hayek], *Silene inflata* Sm.	Caryophyllaceae	4	4	t-s, fl-b	C + R		perennial herb	scapose	0	2000	8	NA	NA	4	7	2	0		B1, E1, H2, I1	EugBrecce, EugGrefra, EugPrapin, EugPrarid, EugRocce, EugRufasco
Silybum marianum (L.) Gaertn.		Asteraceae	4	3	b-r, t-s	C + R		perennial herb	scapose	0	1100	10	10	6	3	5	7	0		E5	EugGrefra, EugRamarb, SinCaorvi, SinIncurb, SinMastaco
Sinapis alba L. *s.l.**		Brassicaceae	2	4	a-p	C		annual herb	scapose	0	1100	8	10	5	3	7	2	0	X	E1, E5, I1	SinCaorvi, SinIncurb
Sinapis arvensis L.*		Brassicaceae	2	4	a-p	C		annual herb	scapose	0	1400	7	5	4	NA	8	6	0		E1, E5, I1	EugGrefra, SinCaorvi, SinIncurb
Sinapis pubescens L.		Brassicaceae	2	2	infl, t-s	C		scrub	scapose	0	1000	8	11	5	3	7	2	0		E5, I1	EugGrefra, EugMagar, EugRocce, SinCaorvi, SinIncurb
Sisymbrium irio L.*		Brassicaceae	2	1	le	R + C		annual herb	scapose	0	800	8	8	5	3	7	5	0		E1, E5, I1, I2, J2	SinIncurb, SinMastaco, SinMuri
Sisymbrium officinale (L.) Scop.*	*Erysimum officinale* L.	Brassicaceae	2	1	infl	R + C		annual herb	scapose	0	2000	8	6	5	4	NA	7	0		E1, E5, I1, I2, J2	SinCaorvi, SinMastaco
Sixalix atropurpurea (L.) Greuter & Burdet	*Scabiosa maritima* L.	Caprifoliaceae	n.a.	1	b-r	C		annual herb	scapose	0	2000	6	8	4	3	NA	2	0		B1, E1, E5, I1	EugBrecce, EugMagar, EugPrarid, EugRocce, EugSand, SinCaorvi
Smilax aspera L.		Smilacaceae	2	3	t-s	C	T	liana	climbing	0	1200	6	10	4	2	5	3	0		F3, F5, G1, G2	EugBofor, EugMagar, EugRamarb, SinIncurb
Smyrnium olusatrum L.		Apiaceae	1	2	t-s, fl-b	R + C	T	perennial herb	scapose	0	800	6	8	3	4	5	9	0		G2, I1	AcqToriflu, EugGrefra, EugRocce, SinCaorvi, SinIncurb
Smyrnium perfoliatum L. *s.l.*	[incl. subsp. *rotundifolium* (Mill.) Hartvig]	Apiaceae	1	2	t-s	R + C	T	perennial herb	scapose	0	1500	6	8	5	4	5	7	0		E2, E5	AcqToriflu, EugRamarb, SinIncurb
Solanum nigrum L.	*Solanum moschatum* C.Presl	Solanaceae	n.a.	1	t-s	C	T	annual herb	scapose	0	900	7	6	5	3	5	7	0		G2, I1, I2, J2	EugGrefra, SinCaorvi, SinIncurb
Sonchus asper (L.) Hill *s.l.*	[incl. subsp. *glaucescens* (Jord.) Ball]	Asteraceae	4	3	a-p	C		annual herb	scapose	0	1500	7	5	NA	4	7	7	0		B3, E1, FB, G2, I1	SinCaorvi, SinIncurb
Sonchus oleraceus L.		Asteraceae	5	5	b-r, le, fl	R (fl) + C		annual herb	scapose	0	1700	7	5	NA	4	8	8	0		E1, G2, I1, I2, J2	SinCaorvi, SinIncurb
Sonchus tenerrimus L.		Asteraceae	5	5	b-r, le, fl	R (fl) + C		annual herb	scapose	0	800	7	8	4	2	5	4	0		E1, I1, I2, J2	EugBrecce, EugGrefra, EugRocce, EugRufasco, SinCaorvi, SinIncurb
Sorbus aria (L.) Crantz		Rosaceae	n.a.	1	fr	R + C		scrub	scapose	0	1200	6	5	5	4	7	3	0		F3, G1	EugBofor, EugRamarb
Sorbus aucuparia L. subsp. *praemorsa* (Guss.) Nyman		Rosaceae	n.a.	1	fr	R + C		tree	scapose	0	2000	6	NA	NA	NA	NA	NA	0	X	F3, G1	EugArbalp, EugBofor, EugRamarb
Sorbus domestica L.		Rosaceae	n.a.	4	fr	R		tree	scapose	0	800	4	7	5	3	8	3	0	X	F3, F5, G1, G2	EugBofor, EugRamarb, SinCaorvi
Stellaria cupaniana (Jord. & Fourr.) Bég.		Caryophyllaceae	2	2	t-s	C		annual herb	caespitose	0	800	6	7	5	4	5	8	0		G2, I1, I2	EugGrefra, EugRocce, SinCalp, SinCaorvi, SinIncurb
Stellaria media (L.) Vill.		Caryophyllaceae	3	2	le, t-s	C		annual herb	caespitose	0	2000	6	NA	NA	4	7	8	0		FB, G2, I1, I2	EugArbalp, EugPrarid, SinCalp
Sulla coronaria (L.) Medik.*	*Hedysarum coronarium* L.	Fabaceae	3	2	t-s	R + C		perennial herb	caespitose	0	1200	7	8	4	5	7	3	0		E1, E2, I1	EugCalan, EugPrapin, EugPrarid, SinCaorvi, SinIncurb
Tamus communis L.	*Dioscorea communis* (L.) Caddick & Wilkin	Dioscoreaceae	3	2	t-s	C		geophyte	climbing	0	1400	5	7	5	5	8	6	0		F3, F5, G1, G2	EugMagar, EugRamarb, SinIncurb
Tanacetum vulgare L. *s.l.*	[incl. *Tanacetum siculum* (Guss.) Strobl]	Asteraceae	n.a.	2	le	R		perennial herb	scapose	0	2700	8	NA	NA	5	NA	5	0		E5	AcqToriflu, EugBofor, EugBrecce, EugRamarb, SinCaorvi, SinIncurb
Taraxacum obovatum (Willd.) DC.		Asteraceae	3	1	b-r	C		perennial herb	rosulate	1500	2000	7	6	4	4	6	6	0		E1, E2, E5	EugPrapin, EugPrarid, SinCaorvi, SinIncurb
Taraxacum officinale Weber ex F.H.Wigg. [Taraxacum sect. *Taraxacum aggregate*]*		Asteraceae	n.a.	1	b-r, bu, fl-b	C		perennial herb	rosulate	0	1700	NA	NA	NA	NA	NA	NA	NA		E1, E2, E5	EugPrapin, EugRamarb, SinCaorvi, SinIncurb, SinMastaco
Teucrium fruticans L.		Lamiaceae	2	1	b-r	C		scrub	caespitose	0	600	11	8	4	2	7	2	0		F5, F6	EugMagar, EugRocce, SinIncurb
Teucrium scordium L. subsp. *scordioides* (Schreb.) Arcang.		Lamiaceae	n.a.	1	a-p	R		perennial herb	scapose	0	1500	7	7	5	8	8	2	0		C3, D5, E2	AcqLapal, AcqToriflu, EugGrefra, EugPrapin
Thymbra capitata (L.) Cav.	*Coridothymus capitatus* (L.) Rchb.f., *Thymus capitatus* (L.) Hoffmanns. & Link	Lamiaceae	n.a.	3	le	R		scrub	caespitose	0	600	11	10	5	2	NA	1	0		F5, F6	EugMagar, EugPrarid, EugRocce, SinIncurb
Thymus spinulosus Ten.		Lamiaceae	n.a.	2	le	R		scrub	caespitose	0	1200	9	8	4	2	2	1	0		E1	EugBrecce, EugMagar, EugPrarid, EugRocce
Tolpis umbellata Bertol.		Asteraceae	2	1	b-r	C		annual herb	scapose	0	1300	11	9	4	2	2	1	0		E1, I1	EugPrarid, SinIncurb

Supplementary Table S1. Critical and updated inventory of the Sicilian NWFP. The asterisks following plant names refer to plant taxa whose native status remains uncertain at the regional level.

Scientific name according to Pignatti et al. (2017-2019)	Synonyms adopted in the consulted literature	Family (APG IV, 2016)	Regional frequency of use as food plant (from Geraci et al., 2018)	Regional frequency of use as food plant (A. La Rosa, pers. data)	Edible parts	Use	Toxic (T) in large amounts or Poisonous (P) if eaten raw without previous treatment	Habit	Growth form	Minimum altitude (m)	Maximum altitude (m)	Ellenberg Indicator Values							CWR	Preferential Habitat - EUNIS	Preferential Habitat - Pignatti et al. (2017-19)
												L	T	C	U	R	N	S			
Tolpis virgata (Desf.) Bertol. *s.l.*	[incl. subsp. *quadriaristata* (Biv.) Giardina & Raimondo and subsp. *grandiflora* (Ten.) Arcang.]	Asteraceae	2	1	b-r	C		perennial herb	scapose	0	1900	11	8	4	2	2	1	0		E1, E5, I1	EugPrarid, SinIncurb
Tordylium apulum L.		Apiaceae	1	1	t-s	R	T	annual herb	scapose	0	1200	11	9	4	2	NA	2	0		E1	EugGrefra, EugMagar, EugPrarid, SinCaorvi, SinIncurb
Tragopogon crocifolius L.		Asteraceae	2	1	le, t-s	C		perennial herb	scapose	600	1700	9	9	4	3	5	3	0		E1, E5	EugPrarid, SinCaorvi, SinIncurb
Tragopogon porrifolius L.	[incl. subsp. *australis* (Jordan) Br.-Bl.]	Asteraceae	2	2	le, t-s, bu, infl	R + C		perennial herb	scapose	0	1000	9	9	5	3	5	3	0	X	E1, I1	EugPrarid, SinCaorvi, SinIncurb
Umbilicus horizontalis (Guss.) DC.	*Umbilicus erectus* DC. , *Cotyledon umbilicus-veneris* L.	Crassulaceae	2	1	le	R		perennial herb	caespitose	0	1200	5	8	4	3	NA	3	0		H3, J2	EugRocce, SinMuri
Umbilicus rupestris (Salisb.) Dandy	*Umbilicus pendulinus* DC.	Crassulaceae	2	1	le	R		perennial herb	caespitose	0	1600	5	8	4	3	NA	3	0		H3, J2	EugRocce, SinMuri
Urospermum dalechampii (L.) F.W.Schmidt	*Tragopogon dalechampii* L.	Asteraceae	4	4	b-r, le	C		perennial herb	scapose	0	1200	8	8	5	3	NA	3	0		E1, I1, J2	EugPrarid, SinCaorvi, SinIncurb
Urospermum picroides (L.) F.W.Schmidt		Asteraceae	4	2	b-r, le	R + C		annual herb	scapose	0	1000	11	9	5	2	NA	2	0		E1, I1, I2, J2	EugPrarid, SinCaorvi, SinIncurb
Urtica dioica L.		Urticaceae	3	1	le	C		perennial herb	scapose	0	2000	NA	NA	NA	6	NA	8	0		E5, I2	EugPrapin, EugRamarb, SinIncurb, SinMastaco
Urtica membranacea Poir.	*Urtica dubia* Forssk.	Urticaceae	3	2	le	C		annual herb	scapose	0	1000	7	8	5	3	6	3	0		G2, I1, I2	EugMagar, EugRamarb, SinCaorvi, SinIncurb, SinMuri
Urtica pilulifera L.		Urticaceae	2	1	le	C		annual herb	scapose	0	1200	7	8	5	3	6	3	0		E5, J2	EugRamarb, SinIncurb, SinMastaco
Urtica urens L.		Urticaceae	3	1	le	C		annual herb	scapose	0	1800	7	6	NA	5	7	8	0		G2, J2	SinCaorvi, SinIncurb, SinMastaco
Valerianella eriocarpa Desv.		Valerianaceae	2	1	a-p	R + C		annual herb	scapose	0	800	11	9	4	2	5	1	0		G2, I1, I2	EugMagar, EugPrarid, EugRocce, SinCaorvi
Valerianella locusta (L.) Laterr.	*Valerianella olitoria* (L.) Pollich	Valerianaceae	2	1	a-p	R + C		annual herb	scapose	0	1400	7	5	5	5	7	NA	0	X	G2, I1, I2	EugMagar, EugPrarid, EugRocce, SinCaorvi
Veronica anagallis-aquatica L.		Plantaginaceae	2	1	le	R		geophyte	scapose	0	1000	7	6	5	9	7	6	0		C3	AcqFapost, AcqLapal, AcqStisori, AcqToriflu
Vicia narbonensis L.*		Fabaceae	n.a.	1	fr	n.a.		annual herb	scapose	0	1000	7	8	5	3	5	5	0		E1, E6, I1	EugPrapin, EugRamarb, SinCaorvi, SinIncurb
Vicia sativa L. *s.l.**		Fabaceae	n.a.	1	fr	n.a.	T (se)	annual herb	climbing	0	1500	5	5	6	NA	NA	NA	0	X	E1, E2, I1	EugGrefra, EugPrapin, EugPrarid, EugRamarb, SinCaorvi, SinIncurb
Viola alba Besser subsp. *dehnhardtii* (Ten.) W.Becker		Violaceae	n.a.	1	fl, le	R + C		perennial herb	rosulate	0	1000	5	8	5	5	7	6	0		E5, F3, G1, G2	EugBofor, EugGrefra, EugPrapin, EugPrarid, EugRamarb, EugRocce
Vitex agnus-castus L.		Verbenaceae	n.a.	1	infl	R	T	scrub	caespitose	0	500	11	11	4	7	NA	2	0		F9	AcqToriflu, EugGrefra, EugMagar, EugRamarb, SinIncurb
Vitis vinifera L. *s.l.*	[incl. subsp. *sylvestris* (C.C.Gmelin) Hegi]	Vitaceae	n.a.	1	fr	R		liana	climbing	0	1200	6	8	5	6	8	6	0	X	F3, F5, F9, G1	AcqToriflu, EugRamarb, SinCaorvi
Xanthium orientale L. subsp. *italicum* (Moretti) Greuter	*"Xanthium strumarium" sensu Auct.*	Asteraceae	2	1	b-r	C		annual herb	scapose	0	800	8	7	5	5	NA	6	0		B1, C3, I1,J2	EugGrefra, EugSand, SinCaorvi, SinIncurb, SinMastaco

Ellenberg Indicator Values
L = Light
T = Temperature
T = Continentality
U = Edaphic humidity
R = Proxy for soil pH
N = Edaphic nutrients
S = Soil salinity

CWR = Crop wild relative
Crop wild relative

Preferential Habitat - EUNIS
A2: Littoral sediment
B1: Coastal dunes and sandy shores
B3: Rock cliffs, ledges and shores, including the supralittoral
C3: Littoral zone of inland surface waterbodies
D5: Sedge and reedbeds, normally without free-standing water
E1: Dry grasslands
E2: Mesic grasslands
E5: Woodland fringes and clearings and tall forb stands
E6: Inland salt steppes
F3: Temperate and mediterranean-montane scrub
F5: Maquis, arborescent matorral and thermo-Mediterranean brushes
F6: Garrigue
F7: Spiny Mediterranean heaths (phrygana, hedgehog-heaths and related coastal cliff vegetation)
F9: Riverine and fen scrubs
FB: Shrub plantations
G1: Broadleaved deciduous woodland
G2: Broadleaved evergreen woodland
G3: Coniferous woodland
H2: Screes
H3: Inland cliffs, rock pavements and outcrops
I1: Arable land and market gardens
I2: Cultivated areas of gardens and parks
J2: Low density buildings

Preferential Habitat (from Pignatti et al., 2017-2019)
AcqFapost: mud, puddles, temporary ponds
AcqLapal: lakes, swamps, marshes
AcqPasal: saltmarshes
AcqStisori: drippings, springs, rivulets, creeks
AcqToriflu: streams, banks and river beds
EugBofor: woodlands and forests
EugBrecce: screes, moraines, volcanic scoriae
EugCalan: clayey badlands
EugCecre: ledges, windy ridges
EugGrefra: riverbeds, landslide slopes
EugMagar: maquis, garrigues
EugPrarid: dry grasslands
EugPrapin: mesic grasslands
EugRamarb: edges, clearigs, deciduous shrubberies
EugRocce: ravine rocks, barks, small outcrops
EugRufasco: rocky faces, cliffs
EugSand: sands
SinCalp: trampled sites
SinCaorvi: crops, vegetable gardens, orchards, vineyards, olive groves
SinIncurb: uncultivated, ruderal and urban habitats
SinMastaco: alms, sheepfolds, manure piles
SinMuri: walls

Edible parts (from Lentini and Venza, 2007, modified)
a-p = aerial parts
b-r = basal rosettes
bu = bulbs and rhizomes
fl = flowers
fl-b = flower buds
fl-n = flower nectar
fr = fruits
infl = inflorescences
le = leaves
ps-fr = pseudo-fruits
ro = roots
se = seeds
st-j = stem juice
t-s = tender shoots

Scale of regional frequency
1: <5% of the considered sites/informants/papers
2: 5-20%
3: 20-50%
4: 50-75%
5: >75%
n.a.: not assigned

Use
R = raw, i.e. fresh, dried, conserved under vinegar or used for drinks
C = cooked, i.e. blanched, boiled, browned, fried, roasted, etc.
S = treated with salt
n.a.: not assigned
