# Supplementary material for: An Updated Checklist of the Sicilian Native Edible Plants: Preserving the Traditional Ecological Knowledge of Century-Old Agro-Pastoral Landscapes
Source: Front Plant Sci. 2020 Apr 29;11:388. doi: 10.3389/fpls.2020.00388 (PMC7201097; doi:10.3389/fpls.2020.00388)
Supplement: Supplementary file 3 [file Table_2.PDF]

Supplementary Table S2. Species mentioned in the Sicilian ethnobotanical literature as NWFP which indeed are not native to Sicily.

Scientific name according to Pignatti et al. (2017-2019)	Synonyms adopted in the consulted literature	Family (Chase et al., 2016)	Status
Agave americana L.		Asparagaceae	Neophyte
Allium schoenoprasum L.		Amaryllidaceae	Archeophyte
Amaranthus retroflexus L.		Amaranthaceae	Neophyte
Antirrhinum majus L.		Plantaginaceae	Archeophyte
Asparagus officinalis L.		Asparagaceae	Archeophyte
Beta vulgaris L.		Amaranthaceae	Archeophyte
Carpobrotus edulis (L.) N.E.Br.		Aizoaceae	Neophyte
Cercis siliquastrum L.		Fabaceae	Archeophyte
Crataegus azarolus L.		Rosaceae	Archeophyte
Cydonia oblonga L.		Rosaceae	Archeophyte
Cyperus esculentus L.		Cyperaceae	Archeophyte
Dysphania ambrosioides (L.) Mosyakin & Clemants	*Chenopodium ambrosioides* L.	Amaranthaceae	Neophyte
Helianthus tuberosus L.		Asteraceae	Neophyte
Isatis tinctoria L.	*Isatis canescens* DC.	Brassicaceae	Archeophyte
Lathyrus sativus L.		Fabaceae	Archeophyte
Lupinus albus L.		Fabaceae	Archeophyte
Morus alba L.		Moraceae	Archeophyte
Morus nigra L.		Moraceae	Archeophyte
Opuntia ficus-indica (L.) Mill.		Cactaceae	Neophyte
Origanum majorana L.		Lamiaceae	Archeophyte
Origanum onites L.		Lamiaceae	Archeophyte
Oxalis pes-caprae L.		Oxalidaceae	Neophyte
Petroselinum crispum Hoffm.		Apiaceae	Archeophyte
Pistacia vera L.		Anacardiaceae	Archeophyte
Prunus dulcis (Mill.) D.A.Webb		Rosaceae	Archeophyte
Punica granatum L.		Lythraceae	Archeophyte
Rhus coriaria L.		Anacardiaceae	Archeophyte
Robinia pseudoacacia L.		Fabaceae	Neophyte
Rumex patientia L.		Polygonaceae	Archeophyte
Ruscus hypophyllum L.		Asparagaceae	Archeophyte
Salvia officinalis L.		Lamiaceae	Archeophyte
Tetragonia tetragonioides (Pall) Kuntze		Aizoaceae	Neophyte
Tropaeolum majus L.		Tropaeolaceae	Neophyte
Wisteria sinensis (Sims) Sweet		Fabaceae	Neophyte
