# Supplementary material for: An Updated Checklist of the Sicilian Native Edible Plants: Preserving the Traditional Ecological Knowledge of Century-Old Agro-Pastoral Landscapes
Source: Front Plant Sci. 2020 Apr 29;11:388. doi: 10.3389/fpls.2020.00388 (PMC7201097; doi:10.3389/fpls.2020.00388)
Supplement: Supplementary file 4 [file Table_3.PDF]

Supplementary Table S3. Wrong, questionable and doubtful records mentioned as edible in the Sicilian ethnobotanical literature and excluded from the current NWFP inventory.

Scientific name according to Pignatti et al. (2017-2019)	Synonyms adopted in the consulted literature	Plant family according to Chase et al. (2016)	Reasons for rejection	Source
Anagyris foetida L.		Fabaceae	Poisonous plant. Its alimentary use is reported for Sardinia (Atzei, 2003) but needs to be confirmed for Sicily. The vernacular name reported by Licata et al. (2016) is usually referred to edible wild peas (*Lathyrus* spp.)	Licata et al. (2016)
Aristolochia sempervirens L.		Aristolochiaceae	Medicinal (and poisonous) plant; its alimentary use has never been reported elsewhere in the whole Mediterranean. The vernacular name reported by Licata et al. (2016) is usually referred to *Silene vulgaris s.l.*	Licata et al. (2016)
Artemisia alba Turra		Asteraceae	Medicinal plant; its use as food plant needs further confirmation. Very rare in central Sicily, it has never been reported to occur on Etna Mt. The vernacular name reported by Licata et al. (2016) is usually referred to *Achillea ligustica*	Licata et al. (2016)
Artemisia arborescens (Vaill.) L.		Asteraceae	Medicinal (and toxic) plant; never used as food in Italy	Provitina (1991); Tuttolomondo et al. (2014b)
Asperula rupestris Tineo		Rubiaceae	Species endemic to the base-rich cliffs and ledges of NW Sicily, its occurrence in NE Sicily has never been recorded	Tuttolomondo et al. (2014a)
Astragalus siculus Biv.	*Astracantha sicula* (Biv.) Greuter ex Reer & Podlech	Fabaceae	Medicinal (and poisonous) plant; its alimentary use has never been reported elsewhere in the whole Mediterranean. Its vernacular name is identical to the one applied to an edible plant, i.e. *Lycium europaeum*: this could the reason for its misidentification	Tuttolomondo et al. (2014b)
Athamanta sicula L.		Apiaceae	Medicinal plant; its use as food plant needs further confirmation	Pasta et al. (2011)
Convolvulus sepium L.	*Calystegia sepium* (L.) R.Br.	Convolvulaceae	Toxic plant; never used as food in Italy	Licata et al. (2016)
Crocus biflorus Mill.		Iridaceae	Very rare in Sicily; probably confused with some more widespread crocuses	Licata et al. (2016)
Descurainia sophia (L.) Webb ex Prantl		Brassicaceae	This species used to grow (probably as a casual alien) in the urban area of Palermo two centuries ago and has never been observed afterwards; probably confused with some other ruderal Brassicaceae	Geraci et al. (2018)
Dittrichia viscosa (L.) Greuter subsp. *viscosa*		Asteraceae	Stinky and resin-rich medicinal plant; never used as food in Italy	Tuttolomondo et al. (2014b)
Elymus repens (L.) Gould subsp. *repens*	*Agropyron repens* (L.) P.Beauv.	Poaceae	Medicinal plant; its use as food plant in Sicily needs further confirmation; famine food in Poland (Łuczaj et al., 2012); the vernacular names reported by Licata et al. (2016) are usally referred to *Cynodon dactylon*	Licata et al. (2016)
Equisetum arvense L.		Equisetaceae	Very rare in Sicily; possibly confused with some more widespread horsetails (*Equisetum* spp.)	Tuttolomondo et al. (2014b); Licata et al. (2016)
Globularia alypum L.		Plantaginaceae	Medicinal (and toxic) plant; never used as food in Italy. The vernacular name reported by Licata et al. (2016) is usually referred to *Artemisia arborescens*. By the way, *Globularia alypum* has never been reported to occur on Etna Mt.	Licata et al. (2016)
Heliotropium europaeum L.		Boraginaceae	Toxic plant; never used as food in Italy	Licata et al. (2016)
Jacobaea erratica (Bertol.) Fourr.	*Senecio aquaticus* Hill. subsp. *erraticus* (Bertol.) Tourlet	Asteraceae	Medicinal (and very poisonous) plant. Its alimentary use has been reported for Tuscany (Corsi and Pagni, 1979); in Sicily it deserves further investigation to be taken for granted.	Geraci et al. (2018)
Lapsana communis L. subsp. *communis*		Asteraceae	Quite rare in Sicily, this species only occurs in the the summergreen broadleaved forests (beech- and oakwoods) of the supramediterranean bioclimatic belt; possibly confused with some more widespread Asteraceae (e.g. *Lactuca muralis*)	Aleo et al. (2013); Geraci et al. (2018)
Leontodon intermedius (Fiori) Huter, Porta & Rigo		Asteraceae	Nobody could be interested on harvesting an extremely rare, rupicolous species; possibly confused with some more widespread *Leontodon*	Geraci et al. (2018)
Marrubium vulgare L.		Asteraceae	This stinky medicinal plant has never been reported as food plant elsewhere in Italy	Licata et al. (2016)
Mentha longifolia (L.) L.		Lamiaceae	This species currently does not occur in Sicily; in the past it has probably been confused with some more widespread wild mints	Pasta et al. (2011)
Muscari botryoides (L.) Mill. *s.l.*		Asparagaceae	No record available on its present or past occurrence in Sicily	Licata et al. (2016)
Nepeta cataria L.		Lamiaceae	Medicinal plant; very rare in Sicily and never used as food in Italy. Probably misidentified	Tuttolomondo et al. (2014b); Licata et al. (2016)
Oloptum miliaceum (L.) Röser & H.R. Hamasha	*Piptatherum miliaceum* (L.) Coss., *Oryzopsis miliacea* (L.) Asch. & Schweinf.	Poaceae	Medicinal plant; never used as food in Italy	Lentini and Venza (2007); Pasta et al. (2011)
Parietaria officinalis L.		Urticaceae	This species does not occur in Sicily; all the past records should be referred to *Parietaria judaica*	Tuttolomondo et al. (2014b)
Polygonatum multiflorum (L.) All.		Asparagaceae	Poisonous plant; its alimentary used has never been reported elsewhere in the whole Mediterranean. The vernacular name reported by Licata et al. (2016) is usually referred to *Ruscus hypophyllum*	Licata et al. (2016)
Polypodium vulgare L.		Polypodiaceae	*Polypodium cambricum* (and not *P. vulgare*) occurs in Sicily. However, the alimentary use of this medicinal (and poisonous) plant has never been documented elsewhere in the whole Mediterranean Basin	Licata et al. (2016)
Prasium majus L.		Lamiaceae	Medicinal plant; never used as food in Italy	Lentini and Venza (2007); Pasta et al. (2011)
Rumex acetosa L. s.l.		Polygonaceae	Absent or extremely rare in Sicily, it has been often confused with *Rumex thyrsoides*. Its occurrence on the island needs to be confirmed	Pasta et al. (2011); Aleo et al. (2013); Geraci et al. (2018)
Ruscus hypoglossum L.		Asparagaceae	This species does not occur in Sicily; previous records probably concern *Ruscus hypophyllum*	Arcidiacono et al. (2007); Pasta et al. (2011)
Ruta graveolens L.		Rutaceae	This species has never been recorded in Sicily; probably misidentified with *Ruta chalepensis*. For this latter, only magic-apotropaic (S. Pasta, *pers. record*) and medicinal uses are known.	Tuttolomondo et al. (2014b)

Supplementary Table S3. Wrong, questionable and doubtful records mentioned as edible in the Sicilian ethnobotanical literature and excluded from the current NWFP inventory.

Scientific name according to Pignatti et al. (2017-2019)	Synonyms adopted in the consulted literature	Plant family according to Chase et al. (2016)	Reasons for rejection	Source
Scabiosa columbaria L. *s.l.*		Caprifoliaceae	This species has never been recorded in Sicily; probably misidentified with *Sixalix atropurpurea*	Licata et al. (2016)
Scorzonera hispanica L. *s.l.*		Asteraceae	This species does not occur in Sicily; it has probably been confused with some native species of *Scorzonera* (e.g. *S. villosa* or *S. hirsuta*)	Licata et al. (2016)
Senecio vulgaris L. *s.l.*		Asteraceae	Medicinal (and poisonous) plant. Its alimentary use has been reported for Latium (Guarrera, 1994); in Sicily it deserves further investigation to be taken for granted	Geraci et al. (2018)
Solanum americanum Mill.		Solanaceae	This species has never been recorded in Sicily; perhaps confused with *Solanum nigrum*	Geraci et al. (2018)
Sonchus arvensis L. *s.l.*		Asteraceae	This species has never been recorded in Italy	Pasta et al. (2011)
Taraxacum campylodes G.E. Haglund		Asteraceae	This species only occurs in Sweden and Norway and has never been recorded in Italy	Licata et al. (2016); Geraci et al. (2018)
Taraxacum caramanicae Lojac.		Asteraceae	The current occurrence and distribution of this probably rare and narrow endemic species needs to be confirmed. In fact, it has never been observed after being described two centuries ago from specimens collected in SE Sicily.	Geraci et al. (2018)
Taraxacum garbarianum Peruzzi, Aquaro, Caparelli & Raimondo		Asteraceae	The current occurrence and distribution of this probably rare and narrow endemic species needs to be confirmed. Nobody could be interested on harvesting such a rare species (Giardina et al., 2007), possibly confused with some more widespread *Taraxacum*	Geraci et al. (2018)
Taraxacum gasparrinii Tineo ex Lojac.		Asteraceae	The current occurrence and distribution of this probably rare and narrow endemic species needs to be confirmed. Nobody could be interested on harvesting such a rare species (Giardina et al., 2007), possibly confused with some more widespread *Taraxacum*	Geraci et al. (2018)
Taraxacum minimum (Guss.) N.Terracc.	*Taraxacum megalorrhizon* (Forssk.) Hand.-Mazz.	Asteraceae	The occurrence of this species in Sicily needs to be confirmed. The species is reported to be a narrow endemic. It has been described two centuries ago and has never been observed hereinafter	Pasta et al. (2011); Licata et al. (2016); Geraci et al. (2018)
Taraxacum siculum Soest		Asteraceae	The current occurrence and distribution of this probably rare and narrow endemic species needs to be confirmed. Nobody could be interested on harvesting such a rare species (Giardina et al., 2007), possibly confused with some more widespread *Taraxacum*	Geraci et al. (2018)
Trifolium phleoides Willd.		Fabaceae	Its alimentary used has never been reported elsewhere in the whole Mediterranean, where only *Trifolium pratense* has been reported as edible	Licata et al. (2016)
Urtica rupestris Guss.		Urticaceae	This endemic species only grows in shady places along some SE Sicilian streams; probably confused with some more widespread nettle species	Tuttolomondo et al. (2014a)
Verbascum sinuatum L.		Scrophulariaceae	Medicinal (and poisonous) plant. Its alimentary use is reported for Sardinia (Atzei, 2003) but needs to be confirmed for Sicily, where it has never been reported before neither in the numerous papers concerning plant uses in Etna region nor elsewhere in the whole island	Tuttolomondo et al. (2014a); Licata et al. (2016)
Xanthium strumarium L. subsp. *strumarium*		Asteraceae	Very rare in Sicily; probably confused with *Xanthium italicum*	Geraci et al. (2018)
